# Phage Display Against 2D Metal–Organic Nanosheets as a New Route to Highly Selective Biomolecular Recognition Surfaces

**DOI:** 10.1002/smll.202406339

**Published:** 2024-11-13

**Authors:** Amelia C. Wood, Edwin C. Johnson, Ram R. R. Prasad, Mark V. Sullivan, Nicholas W. Turner, Steven P. Armes, Sarah S. Staniland, Jonathan A. Foster

**Affiliations:** ^1^ Dainton Building Department of Chemistry University of Sheffield Brook Hill Sheffield S3 7HF UK

**Keywords:** biopanning, metal–organic nanosheet, phage display, quartz‐crystal microbalance, surface plasmon resonance

## Abstract

Peptides are important biomarkers for various diseases, however distinguishing specific amino‐acid sequences using artificial receptors remains a major challenge in biomedical sensing. This study introduces a new approach for creating highly selective recognition surfaces using phage display biopanning against metal–organic nanosheets (MONs). Three MONs (ZIF‐7, ZIF‐7‐NH_2,_ and Hf‐BTB‐NH_2_) are added to a solution containing every possible combination of seven‐residue peptides attached to bacteriophage hosts. The highest affinity peptides for each MON are isolated through successive bio‐panning rounds. Comparison of the surface properties of the MONs and high‐affinity peptides provide useful insights into the relative importance of electrostatic, hydrophobic, and co‐ordination bonding interactions in each system, aiding the design of future MONs. Coating of the Hf‐BTB‐NH_2_ MONs onto a quartz crystal microbalance (QCM) produced a five‐fold higher signal for phage with the on‐target peptide sequence compared to those with generic sequences. Surface plasmon resonance (SPR) studies produce a 4600‐fold higher equilibrium dissociation constant (*K_D_*) for on‐target sequences and are comparable to those of antibodies (K*_D_* = 4 x 10^−10^ m). It is anticipated that insights from the biopanning approach, combined with the highly tunable nature of MONs, will lead to a new generation of highly selective recognition surfaces for use in biomedical sensors.

## Graphical abstract

A new approach for creating highly selective recognition surfaces is reported using phage display biopanning against metal–organic nanosheets (MONs). The highest affinity peptides for three different MONs are isolated through successive biopanning rounds and their structure‐property relationships are investigated. MONs used as recognition surfaces show much higher binding affinities for on‐target peptide sequences with dissociation constants comparable to those of antibodies.

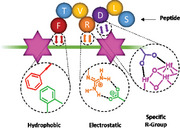

## 1 Introduction

Complex surface structures are responsible for the remarkable degree of biomolecular selectivity found in nature. Macromolecules bind to these bio‐interfaces through multiple weak interactions, with electrostatic and hydrophobic interactions critical in determining selectivity.^[^ ^1^ ^]^ 2D materials represent promising artificial mimics due to their high specific surface areas and accessible functional groups.^[^ ^2^, ^3^ ^]^ A wide range of nanomaterials including transition metal dichalcogenides, MXenes, and graphene have displayed an ability to bind biological macromolecules such as proteins and deoxyribonucleic acid (DNA).^[^ ^4^, ^5^, ^6^ ^]^ However, the simple inorganic structure of these surfaces leads to stochastic functionalization with different binding groups, therefore limiting their selectivity.

Metal–organic framework nanosheets (MONs) are a class of 2D materials that combine organic ligands with metal nodes.^[^ ^7^, ^8^ ^]^ The molecular structure of MONs enables systematic tuning of surface chemistry without changing the overall topology, while their crystalline nature affords a periodic array of recognition sites that promotes multidentate binding.^[^ ^9^ ^]^ Presentation of these functional groups on the surface of the nanosheets, rather than within the pores of a 3D metal–organic framework (MOF), removes any size limitations.^[^ ^10^ ^]^ Early generations of MON‐based biosensors have typically involved fluorescence quenching of dye‐labeled single‐stranded DNA, functionalization with bioreceptors and nanozyme behavior.^[^ ^3^, ^11^, ^12^, ^13^, ^14^, ^15^, ^16^ ^]^ In these examples, much of the sensitivity and selectivity is conferred by other components, such as aptamers and antibodies, enzymes, nanoparticles or other 2D materials.^[^ ^9^ ^]^

To the best of our knowledge, there are currently no studies investigating the interaction of specific sequences of amino‐acids with MONs, although several studies have investigated their interactions with 3D MOFs. Indeed, several MOFs are promising catalysts for the formation and cleavage of peptides, as well as the selective hydrolysis of proteins.^[^ ^17^, ^18^, ^19^, ^20^ ^]^ Parac–Vogt and co‐workers investigated the adsorption of a diverse range of dipeptides onto various Zr‐based MOFs. Extensive screening revealed that affinity depended on hydrophobic, aromatic, and cation–π interactions arising from the hydrophobic/aromatic nature of the linkers and cluster connectivity.^[^ ^21^ ^]^ However, the diversity of both MOFs and peptide sequences and the complexity of their interactions means that identifying strong‐binding combinations for use as highly selective biomolecular recognition surfaces remains a formidable challenge.

**Figure 1:**
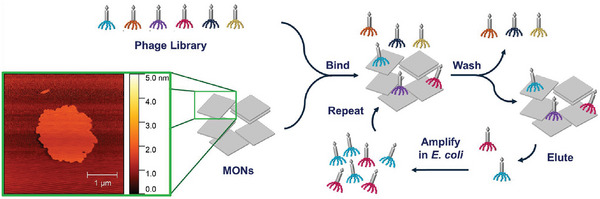
Schematic illustration of phage display against MONs (inset AFM image shows ZIF‐7‐NH_2_), which includes phage incubation with MON to encourage binding, washing, and elution steps, and phage amplification using *E*. *coli*.

Phage display biopanning provides an alternative approach to exploring peptide‐substrate interactions.^[^ ^22^, ^23^ ^]^ A library of bacteriophages presenting various combinations of peptides is incubated with a given target under competitive binding conditions to identify the most strongly binding sequences (**Figure** **1**). In systems other than MOFs, this approach has been used to interrogate antigen‐antibody interactions through epitope mapping as well as to identify suitable peptides for bioinspired mineralization.^[^ ^24^, ^25^, ^26^ ^]^ There is only one report of the use of phage display against MOFs, work by Fan and co‐workers who investigated peptide binding to three stable MOFs (ZIF‐8, Fe‐BTC, and MIL‐53(Al)‐FA). Out of a library of 10^9^ different combinations of twelve amino acid peptides, 2–3 different sequences were found to bind strongly to each MOF with dissociation constants K*_D_* between 10^−5^–10^−7^ m. The authors showed that binding of the peptides to the MOFs could be used to modify their surface properties and inhibit the release of cargo from the MOF pores.^[^ ^27^ ^]^

We hypothesized that biopanning could be used to identify strongly binding peptide sequences to metal–organic nanosheets as a route to creating highly selective MON‐based biomolecular recognition surfaces for use in biomedical sensing applications. We anticipated that the 2D nature of MONs would be advantageous in providing a very high surface area and a single, unique face (top and bottom) to facilitate rapid and highly selective binding. Three different 2D MON systems (ZIF‐7, ZIF‐7‐NH_2,_ and Hf‐BTB‐NH_2_) were selected to explore the effect of subtly different and contrasting components on biomolecular recognition performance. Phage display was utilized to determine which peptides would exhibit strong binding to the surface of each MON out of a library of 1 × 10^13^ possible combinations of 7 amino‐acids. In each case, a single consensus binding peptide sequence with strong affinity to its nanosheet system was identified, highlighting the intrinsic bio‐selectivity of these ultrathin MONs.

The MONs could readily be spin‐coated onto surfaces and MON‐functionalized quartz‐crystal microbalance (QCM) sensors exhibited a five‐fold increase in signal for the selective peptide‐functionalized phage compared to generic peptide sequences for the Hf‐BTB‐NH_2_ system. Subsequently, we investigated the peptide sensitivity of Hf‐BTB‐NH_2_ MONs using surface plasmon resonance (SPR) analysis. This technique indicated a remarkably high equilibrium dissociation constant (*K_D_*) of 4.15 × 10^−10^ m (± 0.32 × 10^−10^) was observed for on‐target interactions which is comparable to naturally occurring biomolecular recognition components such as antibodies.^[^ ^28^ ^]^ Overall, our results demonstrate that specific peptide sequences can exhibit strong selective binding to MONs opening up a new route to the design of next‐generation biomolecular recognition surfaces.

## 2 Results and Discussion

### 2.1 Synthesis and Characterization of Layered MOFs and their Exfoliation to Form MONs

Three different layered MOFs, from two different classes of water‐stable MOFs, were synthesized, and exfoliated to form nanosheets in order to compare how structural differences in their surface affected the binding of peptides.

**Figure 2:**
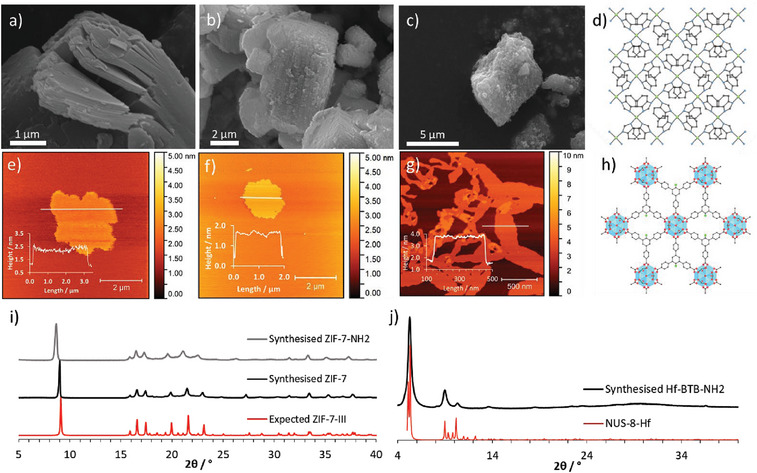
a) Scanning electron microscopy (SEM) image of as‐synthesized ZIF‐7 layered MOF, b) SEM image of ZIF‐7‐NH_2_ layered MOF, c) SEM image of Hf‐BTB‐NH_2_, d) Schematic of ZIF‐7, e) Atomic force microscopy (AFM) image of ZIF‐7 nanosheets, f) AFM image of ZIF‐7‐NH_2_ nanosheets, g) AFM image of Hf‐BTB‐NH_2_ nanosheets h) Schematic of Hf‐BTB‐NH_2_, i) expected X‐ray powder diffraction (XRPD) pattern of ZIF‐7‐III (CCDC‐675375) compared to synthesized ZIF‐7‐III and synthesized ZIF‐7‐NH_2_, j) XRPD pattern of synthesized Hf‐BTB‐NH_2_ compared to NUS‐8‐Hf.

The first system, zeolitic imidazolate framework‐7 (**Figure** **2**d), has previously been reported by a number of groups and used for gas separation, solid‐phase extraction, and membrane filtration.^[^ ^29^, ^30^, ^31^ ^]^ Here we adapted a method developed by Liu et al. and Peng et al. for its synthesis.^[^ ^32^, ^33^ ^]^ Benzimidazole and zinc nitrate hexahydrate were reacted under ambient conditions in dimethylformamide (DMF) to produce a 3D ZIF‐7‐I phase material prior to refluxing in water to induce a hydrothermal phase transition that resulted in the bulk layered phase, ZIF‐7‐III (Figure S1, Supporting Information). The X‐ray powder diffraction (XRPD) pattern of the resulting white microcrystalline powder matched that of the predicted ZIF‐7‐III pattern (Figure 2i) while scanning electron microscopy (SEM) studies also confirmed the formation of the layered material (Figure 2a).

Liquid exfoliation was achieved through ultrasonication of layered ZIF‐7‐III for 2 h in a 1:1 methanol/*n*‐propanol mixture. Ultrathin nanosheets of 0.6 nm mean height and ≈630 nm length were obtained, as indicated by atomic force microscopy (AFM) images (Figure 2e; Figure S18, Supporting Information). Dynamic light scattering (DLS) reported a spherical equivalent diameter of 1063 nm (Figure S21, Supporting Information).

The second MON system, ZIF‐7‐NH_2_, was chosen to explore the effect of subtle differences in the functional groups present on the surface of MONs. We have an ongoing interest in incorporating amine‐functionalized ligands into nanosheet systems to enhance their hydrophilicity and enable post‐synthetic functionalization.^[^ ^34^, ^35^ ^]^ We were inspired by the work of Lopez–Cabrelles et al., who produced an isoreticular series of iron‐based ZIFs incorporating a range of functional groups and sought to create Zn‐based analogs.^[^ ^36^ ^]^ Initial attempts to prepare 100% ZIF‐7‐NH_2_ via the route reported for ZIF‐7 resulted in only partial conversion to ZIF‐7‐NH_2_‐III during reflux of ZIF‐7‐NH_2_‐I in water, resulting in mixed‐phase material (Figure S7, Supporting Information). A mixed ligand system was therefore produced by adding 50 mol.% of 5‐amino‐benzimidazole alongside benzimidazole in the initial step (see Section S3.3, Supporting Information). The XRPD pattern for the final material matched that reported for ZIF‐7‐III, confirming the successful formation of this novel layered material (Figure 2i). Introducing the amine group produced a small shift in the [002] peak, which is attributed to an increase in the inter‐layer distance owing to the incorporation of this bulky group (Figure S8, Supporting Information). The presence of both ligands within the MOF was confirmed by infrared (IR) spectroscopy as well as mass spectrometry analysis of the MOF after acid digestion. ^1^H NMR spectroscopy of the digested material indicated the presence of 20% bim‐NH_2_: this is consistent with a Zn_2_(bim)_3.2_(bim‐NH_2_)_0.8_ composition, as calculated from elemental microanalysis (Figure S5, Supporting Information).

ZIF‐7‐NH_2_ nanosheets were accessed in the same way as for ZIF‐7. AFM studies indicated mean heights and lengths of 1.6 and 400 nm respectively for ZIF‐7‐NH_2_ (Figure 2f; Figure S19, Supporting Information). ZIF‐7‐NH_2_ nanosheets had a relatively broad lateral size distribution reaching lengths of 2 µm but the mean height only ranged from 1 to 5 nm. DLS studies indicated a slight increase in the apparent z‐average diameter for ZIF‐7‐NH_2_ with a narrower particle size distribution compared to ZIF‐7 (Figure S20, Supporting Information).

A third MON system was also evaluated to demonstrate the broad applicability of the approach to different classes of MONs and provide contrasting surfaces for binding studies. A wide range of nanosheets formed from tricarboxylate linkers connected to Zr_6_ or Hf_6_ clusters have been reported for use in catalytic,^[^ ^37^, ^38^, ^39^ ^]^ and photo‐ or radio‐dynamic therapy applications.^[^ ^40^, ^41^, ^42^, ^43^ ^]^ Here Hf‐BTB‐NH_2_, was prepared by modifying the synthetic protocol reported by Ling et al. to introduce Hf_6_ metal clusters.^[^ ^44^ ^]^ Accordingly, hafnium chloride and BTB‐NH_2_ linker were dissolved in DMF then water and formic acid were added as modulating agents. This mixture was heated for 48 h at 120°C and the resulting pale viscous colloidal suspension became a homogeneous suspension after washing. Figure 2j shows the XRPD pattern obtained for the Hf‐BTB‐NH_2_ system compared to Hf‐BTB (NUS‐8) which contains the same key features. The only difference is the introduction of the amine group on the central benzene ring of the BTB linker, see Figure 2h. Further comparative studies by Ling et al. can be found in Figure S13 (Supporting Information), which shows a good match to a simulated pattern for Hf_6_‐BTB MON.^[^ ^44^ ^]^ The bulk material was then ultrasonicated in water (37 KHz, 12 h) to access nanosheets.

The AFM image in Figure 2g shows an example of Hf‐BTB‐NH_2_ nanosheets (average height = 2.7 nm, mean length = 504 nm) with DLS reporting an apparent z‐average diameter of 493 nm (Figures S20 and S21, Supporting Information).

Before phage display was attempted, stability tests were conducted to confirm the structural integrity of the MONs under incubation conditions. Each system was exposed to phosphate buffer saline (PBS) for 1 and 24 h and any structural integrity was determined via XRPD. After incubation for 1 h, the XRPD pattern remained unchanged for each system. After 24 h, only small changes were observed for the ZIFs and no change for Hf‐BTB‐NH_2_ (Figures S23–S25, Supporting Information). Moreover, AFM studies revealed no significant change in particle size after incubation (Figure S26, Supporting Information).

### 2.2 Selection of Peptide‐Functionalized Bacteriophage with High‐Affinity MON Binding

Biopanning studies against the three different MON systems were undertaken to identify the highest affinity peptides. Genetically modified M13 bacteriophages were used which display five identical copies of a 7 amino‐acid peptide.^[^ ^45^, ^46^ ^]^ A library of bacteria phage containing every possible combination of the 7 amino acid sequences (≈1 × 10^13^ combinations) was added to a suspension of each MON, incubated for 1 h, then washed to remove any unbound phage (Figure 1). The pH of the system was adjusted to enable the elution of bound phage from the nanosheets, which were then amplified through the infection of *E. coli*. This protocol constituted one panning round. This process was repeated twice by adding the amplified phage from the previous panning round to create increasingly competitive binding conditions and hence identify peptides with the highest affinities. After the third panning round, twelve phage clones were isolated in each case and their DNA was sequenced to determine those peptide sequences that bound with the highest affinity to each MON system.

The identified peptide sequences are shown in **Table** **1**. Only one dominant peptide sequence was isolated for each MON system after three panning rounds, suggesting high selectivity toward that specific peptide. This contrasts with work on 3D MOFs where Fan et. al. reported 2–3 high‐affinity binding peptides after the final panning round of phage display.^[^ ^27^ ^]^ This is likely explained by the ultrathin nature of the 2D‐MONs which means there is essentially just one type of surface available for binding. This offers a key advantage of 2D MONs over 3D MOFs which have multiple facets that preferentially bind different sequences leading to non‐exclusive binding of peptides.

**Table 1:** Summary of the dominant binding peptides identified by phage display for each MON system.

MON system	MON zeta potential [mV]	MON contact angle [°]	Peptide binding sequence (N’‐C'amidated)	Peptide binding sequence (N’‐C'amidated)	Peptide isoelectric point [pH]	Predicted overall charge in phage display
ZIF‐7	−30.1	149	YNYRNLL	Tyr, Asn, Tyr, Arg, Asn, Leu, Leu	10.15	+ 2
ZIF‐7‐NH_2_	+2.3	141	NNWWAPA	Asn, Asn, Trp, Trp, Ala, Pro, Ala	14.00	+ 1
Hf‐BTB‐NH_2_	−36.9	25	FTVRDLS	Phe, Thr, Val, Arg, Asp, Leu, Ser	10.59	+ 1

### 2.3 Discussion of Peptide Binding Trends

To gain further insight regarding the selection of each preferred peptide sequence, the surface properties of the MONs were compared with those of the consensus peptide.

**Figure 3:**
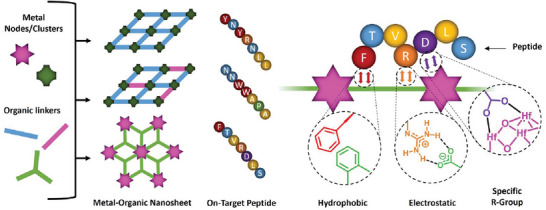
Simplified schematic to show the variety of components used to synthesize three different MON systems where different on‐target binding peptides were identified using phage display and examples of the likely hydrophobic, electrostatic, and specific R‐group affinity interactions.

The binding of the peptide sequences to the periodic array of functional groups on the surface of the MONs is expected to take place through a complex mixture of multiple different intermolecular interactions. Based on related studies,^[^ ^21^ ^]^ we hypothesized that combinations of three different factors were likely to be most important for binding under phage display conditions: 1) hydrophobic interactions, 2) electrostatic interactions, 3) specific R‐group interactions (**Figure** **3**). We therefore generated experimental and calculated data to test the relative importance of each type of interaction which is presented in Table 1.

The hydrophobicity of the MON surfaces was investigated using contact angle measurements and compared to the proportion of hydrophobic/hydrophilic residues within the peptide sequences. Nanosheets were packed onto a glass slide, water drops were added using a micro‐syringe, and the contact angle was measured using a drop shape analyzer image system (Figure S22, Supporting Information). Although there are some limitations to this approach owing to surface roughness effects, clear differences were observed between the two types of nanosheets.^[^ ^47^ ^]^ Hf‐BTB‐NH_2_ nanosheets were considerably more hydrophilic (contact angle = 25°) compared to the ZIF‐7 and ZIF‐7‐NH_2_ nanosheets (contact angles of 149° and 141°, respectively). This correlates with the known properties of these MOF classes.^[^ ^48^, ^49^ ^]^

This trend in hydrophobicity is mirrored by the consensus binding peptides. The ZIF‐7 binding sequence, YNYRNLL, contains several hydrophobic tyrosine and leucine residues, while the ZIF‐7‐NH_2_ peptide sequence, NNWWAPA, contains multiple hydrophobic tryptophan and alanine residues. Aromatic Tyr and Trp residues may also participate in *π–π* interactions with the electron‐rich aromatic benzimidazole ligands of ZIF‐7.^[^ ^50^, ^51^ ^]^ In contrast, the Hf‐BTB‐NH_2_ sequence, FTVRDLS, contains a broader range of amino‐acids, with only three hydrophobic residues (phenylalanine, valine, and leucine) alongside hydrophilic residues such as threonine and serine, as well as anionic aspartic acid and cationic arginine residues. This suggests that hydrophobic binding effects are most likely dominant for the ZIF nanosheets but are much less important for the more polar Hf‐BTB‐NH_2_ nanosheets.

To investigate the importance of electrostatic interactions, aqueous electrophoresis studies of the nanosheets were undertaken in the presence and absence of each peptide. Zeta potentials of the three nanosheet systems were determined in dilute PBS. ZIF‐7 and Hf‐BTB‐NH_2_ nanosheets exhibited moderately negative zeta potentials of −30.1 and −36.9 mV, respectively. Thus, the surface chemistry of the ZIF systems is dominated by the anionic imidazole ligands, rather than the zinc cations. The negative zeta potential for Hf‐BTB‐NH_2_ most likely indicates the presence of surface hydroxyl and phosphate groups, which would confer an overall anionic charge. A weakly positive zeta potential of +2.3 mV was observed for ZIF‐7‐NH_2_ and was attributed to the presence of surface amine groups. Such a low zeta potential is often associated with an unstable colloidal suspension. However, no sedimentation was observed over the timescale of the experiment and the zeta potential data proved to be reproducible.

Amino‐acid side‐chain and N‐terminus pKa values were used to calculate the isoelectric point of the binding peptide and to predict the overall charge under phage display (PD) conditions (see section S5.3, Supporting Information). All three preferred peptides sequences are cationic. The ZIF‐7‐NH_2_ sequence contained no ionizable R‐groups but has a +1 charge from the terminal amine group. Both ZIF‐7 and Hf‐BTB‐NH_2_ contained arginine which confers an additional +1 charge. This gives the sequence for ZIF‐7 an overall charge of +2, but is balanced out by an aspartic acid group in the case the case of Hf‐BTB‐NH_2_ to give a zwitterionic peptide with an overall charge of +1.

The change in zeta potential (ΔZP) was determined upon MON incubation with excess binding peptide (Figure S30, Supporting Information). A large ΔZP is observed for ZIF‐7 (+56.2 mV) and Hf‐BTB‐NH_2_ (+ 40.0 mV) but a rather smaller difference is observed for ZIF‐7‐NH_2_ (+ 20.9 mV). Thus, electrostatic interactions seem to be important for the binding of cationic peptides to the anionic surfaces of ZIF‐7 and Hf‐BTB‐NH_2_, but this effect is less significant for peptide binding to ZIF‐7‐NH_2_.

Finally, we considered other possible interactions from R‐groups present in the consensus peptide sequences. In particular, the inclusion of negatively charged aspartic acid residues in the consensus peptides binding to the overall anionic surface of Hf‐BTB‐NH_2_ nanosheets seems counterintuitive. Lan et al. demonstrated that various amino‐acids can bind to the surface of Hf_12_‐cluster nanosheets via ligand exchange of capping formate groups by co‐ordination of carboxylic acids.^[^ ^52^ ^]^ In principle, the anionic carboxylate form of aspartic acid (which has a side‐chain *p*K_a_ of 3.90) may similarly displace the capping formate ligand (*p*K_a_ 3.75) to bind to the Hf clusters explaining its presence in the consensus peptide despite the similar charges.

Greater detail about the relative importance of the presence and position of specific amino‐acids within the strongly binding peptide sequences can be obtained by identifying other, not quite so strongly binding sequences, present in earlier panning rounds. Peptides from the first panning round of Hf‐BTB‐NH_2_ were therefore sequenced and are shown in **Table** **2**. Interestingly, the sequence VRD was conserved in four of the six peptides isolated from the first panning round, with RDL conserved in three out of six peptides. The conserved nature of this sequence indicates that multiple amino‐acids are involved in cooperative binding interactions, rather than individual amino acids acting in isolation. Furthermore, it indicates that this combination of an anionic carboxylate group adjacent to a cationic arginine group and a hydrophobic valine/leucine group within the consensus peptide sequence may be key to its strong binding.

**Table 2:** Summary of the binding peptides identified by phage display in panning rounds one and three against Hf‐BTB‐NH2 with similarities highlighted in yellow.

MON System	Panning round	Peptide binding sequence (N’‐C'amidated)	Peptide isoelectric point [pH]	Predicted overall charge in phage display
Hf‐BTB‐NH_2_	3	FTVRDLS	10.59	+ 1.0
	1	FTVRDLS	10.59	+ 1.0
	1	FPVRDLS	10.59	+ 1.0
	1	STVRDFS	10.57	+ 1.0
	1	SPVRDNW	10.57	+ 1.0
	1	YPERDLC	6.19	−0.1
	1	ISPHPGS	14.00	+ 1.1

For the sixth sequence, ISPHPGS, only the final serine is conserved compared to the consensus peptide. In this case, arginine is replaced by a cationic histidine residue in the central fourth position, with the latter flanked by hydrophobic proline residues but no residues with coordinating carboxylate R‐groups. The constrained proline rings mean that this peptide is likely to have a significantly different set of interactions with the MON surface. Presumably, this produces a local minimum on the energy binding landscape and indicates a range of other strong binding sequences may be possible.

A search amongst the >233 million proteins within the Universal Protein Knowledgebase (UniProtKB) for the strongly binding 7 amino‐acid sequences identified in this study showed they appear in multiple known peptides. For example, FTVRDLS appears in 254 proteins including 74 with known catalytic activity, 31 signal peptides, and 82 transmembrane proteins. These MONs are therefore promising recognition surfaces to selectively bind biologically important proteins where the relevant peptide sequence is displayed on the outside.

Many other peptides of biomedical significance will have related sequences. Understanding from systematic studies such as these offer the potential of reverse engineering MONs with the correct properties to target these. Some design principles that emerge from these studies are relatively intuitive, for example, that cationic peptides would bind to anionic peptide surfaces, that peptides with more hydrophobic amino‐acid groups favor more hydrophobic peptides and that the inclusion of co‐ordinating R‐groups may favor binding to MONs with labile metal sites. Identification of conserved sequences such as VRDL are also particularly informative as the probability that these sequences of amino‐acids will be present and accessible in biomedically important peptides is much higher than for longer sequences. None of the consensus peptides identified are simply the most hydrophobic, most charged, most co‐ordinating, or contain consensus sequences and binding must therefore be the result of a subtle combination of all of these interactions as well as their spacing and orientation as determined by their sequence. It is possible that the consensus peptides obtained from biopanning are influenced by factors other than just pure binding affinity, for example their stability or how easy it is for bacteriophage to amplify particular amino‐acid sequences. Nevertheless, these high affinity “hits” provide valuable data that aids understanding sequence‐surface binding relationships and so aid the design of new MONs to target biomedically interesting peptide sequences.

### 2.4 MON‐Coated QCM Sensors for the Detection of Peptide‐Displaying Bacteriophage

Over the past decade, various quartz crystal microbalance (QCM) based biosensors have been developed that offer high sensitivity and short detection times.^[^ ^53^, ^54^, ^55^, ^56^, ^57^ ^]^ A typical QCM biosensor contains either natural or synthetic biomolecular recognition components such as antibodies or molecularly‐imprinted polymers (MIPs) coated on a QCM surface. Natural receptors suffer from instability while technical issues associated with MIPs lead to sub‐optimal detection limits.^[^ ^55^ ^]^ We hypothesized that 2D‐MONs might offer an alternative biomolecular recognition platform because they constitute a highly stable periodic array of active sites and can be easily processed to form thin films. Examples of 3D‐MOF‐based QCM sensors for vapor/gas detection have been reported but such coatings are relatively thick (≈ 1 µm).^[^ ^58^, ^59^ ^]^ A 2D‐MON‐based QCM sensor has been produced for ammonia detection but as far as we are aware there have been no other studies involving MON‐based QCM sensors.^[^ ^60^ ^]^ QCM was used to assess intrinsic structural changes within MON‐based water filtration membranes but was not utilized as a sensor.^[^ ^61^ ^]^ Xie et al. designed a covalent‐organic framework nanosheet‐based QCM sensor to detect miRNA but in this case, the nanosheet was simply employed to enhance signal generation in a complex probe DNA system.^[^ ^62^ ^]^

One major advantage of a QCM sensor is its sensitivity: detectable changes in adsorbed mass are of the order of one nanogram per cm^2^. The simplest model relating ∆*f* to *m* is the Sauerbrey equation,^[^ ^63^ ^]^ which is used to calculate the mass of the adsorbate.

**Figure 4:**
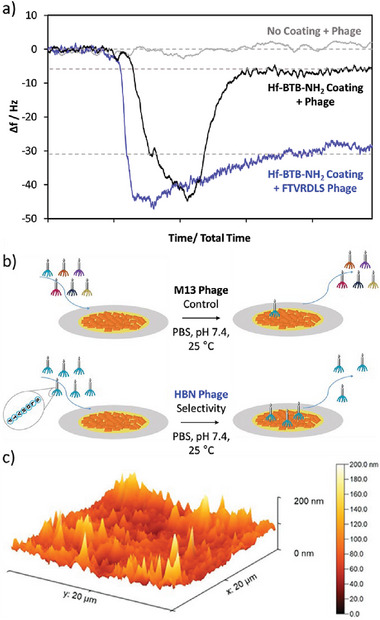
a) Change in frequency, Δ*f*, observed for a silica QCM sensor with no coating (grey) and a ≈100 nm Hf‐BTB‐NH_2_ coating after exposure to an aqueous solution of M13 phage in PBS at pH 7.4. Δ*f* was also determined for a ≈100 nm Hf‐BTB‐NH_2_‐coated silica QCM sensor after exposure to a FTVRDLS‐functionalized phage in PBS at pH 7.4, b) Schematic illustration of the difference between exposing this Hf‐BTB‐NH_2_‐coated QCM sensor to phage and FTVRDLS phage, c) AFM image of the Hf‐BTB‐NH_2_ coated silica QCM used to determine the mean coating thickness of ≈100 nm.

Silica‐based QCM substrates were coated with MONs to investigate their performance in sensing various viruses at a constant flow rate of 0.018 mL min^−1^. The Hf‐BTB‐NH_2_ system was selected as it was the most stable in PBS (Figure S25, Supporting Information) and minimal signal drift was observed. This QCM sensor had a Hf‐BTB‐NH_2_ coating of a mean thickness of ≈100 nm, see **Figure** **4**c. Phage‐displaying FTVRDLS, the consensus peptide for Hf‐BTB‐NH_2_, was compared with generic 7‐mer peptides as a control to assess the extent of non‐specific binding.

QCM data obtained using a sensor with no MON coating (grey) and two sensors coated with Hf‐BTB‐NH_2_ MON (black and blue) are shown in Figure 4. Uncoated and MON‐coated QCM sensors were exposed to control phage (grey and black). The uncoated QCM sensor showed no Δ*f* and the MON‐coated sensor with generic phage exhibited only a small Δ*f*, which corresponds to an adsorbed amount of just 0.38 mg m^−2^. These observations indicate a weak interaction between the MON surface and the generic phage but no detectable interaction with the uncoated silica sensor control. In contrast, the consensus phage (displaying FTVRDLS) exhibited a much greater Δ*f* corresponding to an adsorbed amount of 1.87 mg m^−2^; a five‐fold increase from the control phage. This confirms that phage display identified a selective peptide and that MON‐based QCM sensors can be readily constructed.

### 2.5 Binding Performance of Peptides to Synthesized Hf‐BTB‐NH_2_ Nanosheets

QCM studies provided strong evidence of highly selective binding to the phage. However, investigations of individual peptides were unsuccessful owing to the relatively high flow rates required for the set‐up. To gain a better understanding of the recognition capabilities of the nanosheets for isolated peptides not attached to phage, zeta potential, and surface plasmon resonance (SPR) measurements were undertaken to determine the dissociation constant (*K_d_*) and equilibrium dissociation constant (*K_D_*) for the on‐target and off‐target peptides to Hf‐BTB‐NH_2_.

A saturation binding experiment was conducted in which zeta potential measurements were recorded for a stock MON solution (1 mg mL^−1^ in 1.37 mmol PBS w.r.t. NaCl) incubated with various peptide concentrations (Figure S31, Supporting Information). The *K_d_* for FTVRDLS binding to Hf‐BTB‐NH_2_ was calculated to be 74 ± 22 µm. Off‐target binding studies of YNYRNLL and NNWWAPA against Hf‐BTB‐NH_2_ were also conducted, with the *K_d_* determined to be 285 ± 23 and 1393 ± 200 µm respectively. This corresponds to an ≈4 and 19‐fold increase in the dissociation constant relative to the on‐target binding of FTVRDLS. This is consistent with the phage display identified 12‐mer peptide binding studies against MOFs reported by Fan et. al., who found all values lying within the micromolar range.^[^ ^27^ ^]^ The smaller difference in *K_d_* for YNYRNLL most likely reflects similarities between these arginine‐bearing peptides, which leads to strong binding to the anionic MON surfaces. Hence Hf‐BTB‐NH_2_ shows a high degree of selectivity for the consensus peptide identified through the biopanning studies, which makes it a promising candidate for use as a sensitive peptide recognition surface.

SPR analysis was used to determine the overall equilibrium dissociation constant (*K_D_*) of Hf‐BTB‐NH_2_ nanosheets toward these peptides and the data is summarized in **Table** **3**. Covalent attachment of the nanosheets to the functionalized gold surface was achieved through a three‐step process. A planar polyethylene glycol/carboxyl‐coated Au chip was activated using NHS and EDC. This was followed by the addition of a dispersion of the nanosheets in running buffer where the activated carboxylic acid groups reacted with the amine‐grous on the surface of the MONs. Finally, a quenching solution of ethanolamine was used to deactivate any unreacted carboxyl groups and wash away any unbound nanosheets. Figure S32 (Supporting Information) shows a change in refractive index consistent with MON functionalization on the chip surface. A monolayer is expected to form, as nanosheets not directly covalently bound to the surface will be washed away.

**Table 3:** Calculated equilibrium dissociation constant (nM) of imprinted materials. All experiments performed under ambient conditions *n* = 3.

	*K_D_* [sM]
Target Peptide‐FTVRDLS	4.15 × 10^−10^ (±0.32 × 10^−10^)
Off‐target Peptide‐YNYRNLL	2.71 × 10^−7^ (±0.48 × 10^−7^)
Off‐target Peptide‐NNWWPA	1.91 × 10^−6^ (±0.27 × 10^−6^)

The SPR sensorgrams (Figure S33, Supporting Information) show the interactions of the target peptide FTVRDLS at five different concentrations with the Hf‐BTB‐NH_2_ nanosheets. To study cross‐reactivity and non‐specific binding to the nanosheets were also investigated with non‐target peptides YNYRNLL and NNWWAPA were also examined, Tween 20 surfactant (0.01%) was added to the running buffer to minimize non‐specific binding. Experiments were repeated in triplicate and the SPR curves were fitted to a 1:1 interaction model. Plotting the maximum signal (µRIU) from these curves against concentration allowed the limit of detection to be estimated as 0.39 nm (376 ng L^−1^) (Figure S34, Supporting Information).

The *K_D_* value of the interaction between target peptide FTVRDLS and the nanosheet has been calculated at 0.415 nm (Table 3). This shows that Hf‐BTB‐NH_2_ nanosheets exhibit extremely high affinity toward the target peptide FTVRDLS and the strength of this specific interaction is comparable to natural recognition components such as antibodies.^[^ ^28^ ^]^ The interaction of this nanosheet with non‐target peptides, produced *K_D_* values of 271 and 1910 nm for YNYRNLL and NNWWAPA, respectively. This suggests a high degree of selectivity and specificity toward the target peptide, with an ≈650 and 4600‐fold improvement in affinity compared with off‐target YNYRNLL and NNWWAPA, respectively.

The nanosheets immobilized on the SPR‐chips were used for a total of 45 different experiments with no‐observed degradation indicating a high level of stability and re‐usability. This provides further evidence that Hf‐BTB‐NH_2_ nanosheets are excellent candidates as biomolecular recognition components within sensors.

## 3 Conclusion

Achieving high selectivity for a specific peptide sequence amongst the myriads of possibilities is a formidable challenge with enormous potential for the development of next‐generation biosensors. Randomly combining different MONs with biomedically important peptides through a conventional trial‐and‐error process is unlikely to identify high‐affinity combinations due to the sheer number of possible amino‐acid sequences and MONs. We therefore exploited phage display biopanning as an alternative route to identifying strongly binding peptides and enable us to gain insights into sequence/surface relationships that will allow the design of new MONs for biosensing applications.

Three ultrathin MON systems were synthesized with similar, ZIF‐7 and ZIF‐7‐NH_2_, and contrasting, Hf‐BTB‐NH_2_, chemical structures. Phage display biopanning allowed simultaneous screening of a library of 1 × 10^13^ possible combinations of 7 amino‐acids to identify the strongest binding peptide sequence for each of the three different MONs. In contrast to previous biopanning studies on 3D MOFs, only a single consensus peptide sequence was identified in each case which is attributed to the MONs surface comprising only a single facet. The 7 amino‐acid sequences identified in the study are present in many biologically relevant proteins making the MONs promising candidates for acting as highly selective recognition surfaces for proteins where these sequences are displayed on the outside.

Comparison of zeta potential data and contact angle measurements obtained for these MONs with calculated properties for the peptide sequences indicated that a subtle combination of interactions was important for strong peptide‐MON binding. Hydrophobic residues were key to binding both ZIF systems, with an additional electrostatic contribution from positively charged residues in the case of ZIF‐7 which had a negative surface charge in contrast to the slight positive charge of ZIF‐7‐NH_2_. Hydrophobicity was much less important in binding to the more polar Hf‐BTB‐NH_2_ system with a combination of electrostatic attraction and coordination by aspartic acid key to binding. The sequence VRDL was highly conserved amongst six strongly binding peptides from the 1st panning round confirming that the sequence of amino‐acids, rather than just the presence of particular R‐groups, is key to determining binding. Insights such as these, in combination with further experimental and computational studies looking at libraries of other structurally related metal–organic nanosheets, will allow a detailed understanding of sequence‐structure relationships to be built up. and aid the design of future metal–organic materials able to target biomedically significant peptides.

A QCM‐MON sensor functionalized with Hf‐BTB‐NH_2_ was constructed as a model for binding and a five‐fold increase in selectivity was demonstrated for phage displaying the consensus peptide compared to phage displaying generic peptides. Furthermore, SPR studies confirmed that the FTVRDLS consensus sequence binds up to 4600 times more strongly to Hf‐BTB‐NH2 than to the off‐target peptide sequences with values comparable to those achieved by antibodies (*K_D_* = 4 × 10^−10^ m). The impressive affinities and selectivities achieved by these systems demonstrate the potential of MONs as recognition surfaces which, combined with their low cost, high stability and the ease with which different surfaces can be coated make them promising candidates for use in biosensors.

## Conflict of Interest

The authors declare no conflict of interest.

## Author Contributions

A.C.W. undertook the synthesis and characterization of the ZIF‐7‐based MONs, biopanning and peptide binding studies, and MON‐QCM sensors as well as analyzing the data and drafting the manuscript. E.C.J. provided training on QCM. and added data discussion and editing of the manuscript. R.R.R.P. synthesized the Hf‐BTB‐NH_2_ MONs and edited the manuscript. M.V.S. undertook S.P.R. experiments and aided data analysis. N.W.T. aided data discussion and editing of the manuscript. S.‐P.A. provided the QCM equipment and edited the manuscript. S.S.S. co‐supervised the project and edited the manuscript. J.A.F supervised the project, and aided with data discussion and drafting and editing of the manuscript.

## Supporting information



Supporting Information

## Data Availability

The data that support the findings of this study are available in the supplementary material of this article.
