# Supplementary material for: Phage Display Against 2D Metal–Organic Nanosheets as a New Route to Highly Selective Biomolecular Recognition Surfaces
Source: Small. 2024 Nov 13;21(29):2406339. doi: 10.1002/smll.202406339 (PMC12288780; doi:10.1002/smll.202406339)
Supplement: Supplementary file 1 — Supporting Information [file SMLL-21-2406339-s001.docx]

**Phage display against two-dimensional metal-organic nanosheets as a new route to highly selective biomolecular recognition surfaces**

Amelia C. Wood, Edwin C. Johnson, Ram R. R. Prasad, Mark V. Sullivan, Nicholas W. Turner, Steven P. Armes, Sarah S. Staniland, Jonathan A. Foster

Department of Chemistry, University of Sheffield, Brook Hill, Sheffield S3 7HF

*KEYWORDS. Phage Display, Metal-Organic Nanosheet, Biopanning, Quartz-crystal microbalance*

[General Details 106](#_Toc162541197)

[2.1 Materials 106](#_Toc162541198)

[2.2 Analytical Procedures 106](#_Toc162541199)

[Synthesis of Metal-Organic Materials 108](#_Toc162541200)

[3.1 Synthesis of ZIF-7-III MOF 108](#_Toc162541201)

[3.2 Characterisation of ZIF-7-III MOF 108](#_Toc162541202)

[3.3 Synthesis of ZIF-7-NH_2_-III MOF 110](#_Toc162541203)

[3.4 Characterisation of ZIF-7-NH_2_-III MOF 111](#_Toc162541204)

[3.5 Synthesis of Hf-BTB-NH_2_ 115](#_Toc162541205)

[3.6 Characterisation of Hf-BTB-NH_2_ 115](#_Toc162541206)

[MON Synthesis and Characterisation 118](#_Toc162541207)

[4.1 Exfoliation of ZIF-7 and ZIF-7-NH_2_ to access nanosheets 118](#_Toc162541208)

[4.2 Exfoliation of Hf-BTB-NH_2_ to access nanosheets 118](#_Toc162541209)

[4.3 Characterisation of ZIF-7, ZIF-7-NH_2_ and Hf-BTB-NH_2_ nanosheets 118](#_Toc162541210)

[Phage Display 126](#_Toc162541211)

[5.1 Panning Round Data 127](#_Toc162541212)

[5.2 Zeta Potential 127](#_Toc162541213)

[5.3 QCM binding studies 129](#_Toc162541214)

[5.4 SPR binding studies 129](#_Toc162541215)

[5.5 Isoelectric point calculations 132](#_Toc162541216)

[References 132](#_Toc162541217)

General Details

1. Materials

Commercial solvents, reagents and spectroscopic grade deuterated solvents were used as purchased without further purification, as listed: 5-aminobenzimidazole (fluorochem), benzimidazole (99 %, Alfa Aesar), deuterium oxide (99.9 atom % D, Sigma-Aldrich), dimethylformamide (≥99 %, Fisher), dimethyl sulfoxide-d6 (99.5 atom % D, Sigma-Aldrich), deuterium chloride solution (35 wt. % in D_2_O, ≥99 atom % D, Sigma-Aldrich), ethanol (≥99.8 %, Fisher), formic acid (98+%, Acros Organics), hafnium chloride (98+ %, Alfa Aesar), n-propanol (SLS), sodium deuteroxide (40 wt. %, Sigma-Aldrich), zinc nitrate hexahydrate (Zn(NO_3_)_2_·6H_2_O, 98 %, Acros Organics)

7aa-Phage display kit (New England Biolabs), Miniprep Kit (Thermo Scientific GeneJET Plasmid)

1-Ethyl-3-(3-dimethylaminopropyl)carbodiimide (EDC), glycine, (BIS), *N*-hydroxysuccinimide (NHS), were purchased from Sigma-Aldrich (Poole, Dorset, UK).

Dipotassium phosphate, disodium phosphate, potassium chloride, sodium acetate, sodium chloride, and Tween 20 were all purchased from Fisher Scientific UK (Loughborough, Leicester, UK).

Planar Polyethylene Glycol/Carboxyl Surface Sensor chips were purchased from Reichert Technologies Life Sciences, Buffalo, New York, USA.

The SPR running buffer (PBST) was a phosphate buffered saline made at 10 mM, pH 7.4, supplemented with 0.01 % (v/v) Tween 20. Tween 20 is included to reduce non-specific binding during rebinding studies. Regeneration buffer was 10 mM Glycine-HCl at pH 2.

1. Analytical Procedures

Elemental analysis was performed by the microanalytical service at the Department of Chemistry, University of Sheffield using a Vario MICRO Cube in an atmosphere of pure O_2_. Elemental CHN contents are determined to a tolerance of ± 0.5 % for organometallics.

FT-IR spectra were recorded using a Perkin Elmer Spectrum 100 FT-IR spectrophotometer, equipped with a SenseIR diamond ATR module. Samples were analysed without further preparation, and spectra were obtained in reflectance mode between 4000 – 400 cm^-1^, using 12 scans with a spectral resolution of 1 cm^-1^.

NMR spectra were recorded at 300 K using a Bruker AV 400 spectrometer with a 5mm solution state probe at 400 MHz (1H). Supramolecular frameworks were digested prior to submission, using DCl (30 µL) and DMSOd6 (750 µL) for the ZIF systems and NaOD (100 µL) and D_2_O (750 µL) for Hf-BTB-NH_2_ with ultrasonication (24 h). NMR spectra were processed using MestreNova. Mass spectra were recorded directly from NMR solutions using an Agilent 6530 QTOF LC-MS in negative ionisation mode.

Powder X-ray diffraction (XRPD) data were collected using a Bruker-AXS D8 diffractometer using Cu Kα (λ=1.5418 Å) radiation and a LynxEye position sensitive detector in Bragg Brentano parafocussing geometry using a packed glass capillary or a flat silicon plate.

Thermogravimetric analyses (TGA) were performed using a Perkinelmer pyris 1 instrument. Approximately 4 mg of sample was weighed into a ceramic pan, held under nitrogen flow of 20 cm^3^ min^-1^ at 25 °C for 10 minutes to purge the sample and allow for equilibration, then ramped to varying end temperatures (see individual traces for details) at 1 °C min^-1^ . The samples were then held at the final temperature for 10 minutes.

Dynamic light scattering (DLS) data were collected using a Malvern Zetasizer Nano Series particle size analyser, using a He-Ne laser at 633 nm, operating in backscatter mode (173 °). Samples were equilibrated at 298 K for 60 s prior to analysis. Zeta potential data were collected using the same instrument in zeta potential mode, using disposable polycarbonate capillary electrophoresis cells and according to the Smoluchowski method. All materials were dispersed in diluted PBS (pH 7.4) to 1.37 mmol with respect to NaCl. Samples were incubated with peptide for exactly 1 h before    measurements were taken. Contact angle measurements were obtained using a Drop Shape Analyzer using the Sessile drop method.

Atomic force microscopy (AFM) images were recorded using a Bruker Multimode 5 Atomic Force Microscope, operating in soft-tapping mode in air under standard ambient temperature and pressure, fitted with Bruker OTESPA-R3 silicon cantilevers operated with a drive amplitude between 5-20 mV and resonance frequency of ~236 kHz. ZIF MON solutions were prepared (0.2 mg / mL, methanol-propan-1-ol, 1:1 volume) and Hf-BTB-NH_2_ MON solutions (2.5 mg / mL ethanol, 2 drops into 20 mL vial of ethanol and vigorous shaking). AFM samples were prepared by drop-casting 10 µL drops of suspension onto the centre of freshly cleaved mica sheets heated to 120 °C on a hot plate. These sheets were stuck to stainless steel, magnetic Agar scanning probe microscopy specimen discs. Images were processed using Gwyddion software.

Scanning electron microscopy (SEM) samples were prepared by loading powdered samples onto carbon sticky tabs placed on aluminium SEM sample stubs, coated with approximately 20 nm gold using an Edwards S150B sputter coater and loaded into a TESCAN VEGA3 LMU SEM, operated at 15 keV. Images were collected at 10,000x magnification using the secondary electron detector.

For peptide sequencing, phage plasmid was extracted using a miniprep kit. The samples were sent off to GENEWIZ for sanger sequencing.

Synthesis of Metal-Organic Materials

1. Synthesis of ZIF-7-III MOF

Zinc nitrate hexahydrate (755 mg, 2.54 mmol) and benzimidazole (1.92 g, 16.28 mmol) were dissolved in DMF (250 mL, 3.25 mol). After stirring for 1 h, the solution is kept statically at room temperature for 72 h. ZIF-7 nanoparticles were then collected by centrifugation and thoroughly washed with methanol and dried at 50 °C overnight. Then subsequently dried at 120 C for 48 h in a vacuum oven. The obtained ZIF-7-I was redispersed in distilled water at a concentration of 0.5 wt % and then refluxed at 100 C for 24 h. The turbid mixture was washed with distilled water once and methanol three times, and then dried at 50 C overnight to obtain ZIF-7-III (486 mg, 0.805 mmol, 42 %) as a white powder. Elemental Analysis %: C 56.1, H 3.3, N 18.7 Found Mass %: C 56.21, H 3.51, N 18.6; (ESI-NEG): [M]^+^ 119.1 (benzimidazole). ^1^Η ΝΜR (DCL/DMSO-d_6_): 9.66 (s, 1Η), 7.92 – 7.83 (m, 2Η), 7.63 – 7.55 (m, 2Η). Phase purity confirmed by XRPD with a comparison to the CCDC (675375) powder pattern for ZIF-7-III.

1. Characterisation of ZIF-7-III MOF

XRPD


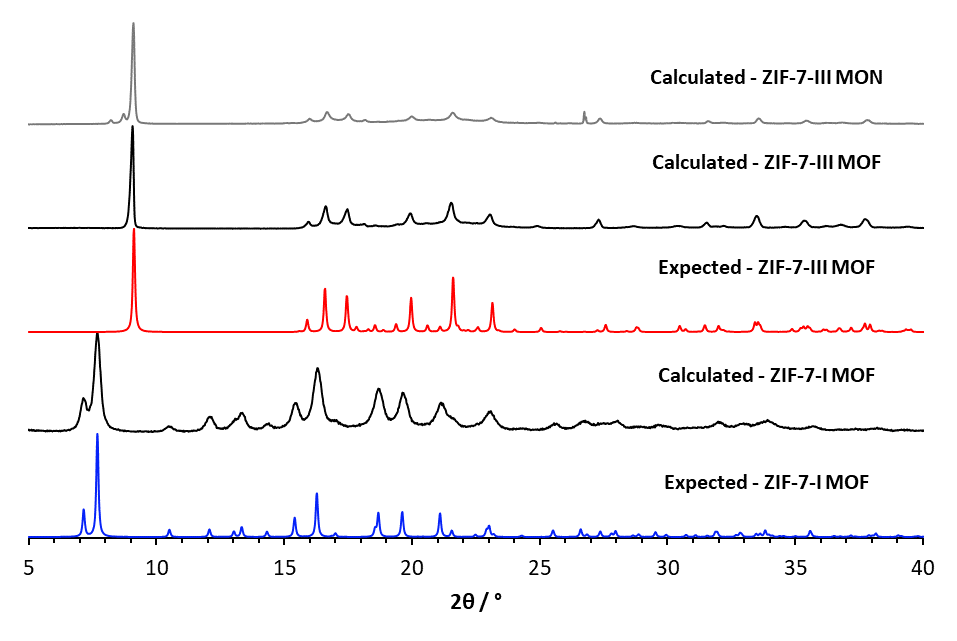


Figure S1: XRPD of the different phases of ZIF-7 MOF and 2D MON. The expected pattern of ZIF-7-I MOF found in CCDC (602541) (blue) with comparison to the calculated ZIF-7-I MOF (black). The expected pattern of ZIF-7-III MOF found in CCDC (675375) (red) with comparison to the calculated ZIF-7-III MOF (black) and the calculated ZIF-7-III MON (grey).

ATR-IR

Figure S2: ATR-IR spectrum of ZIF-7


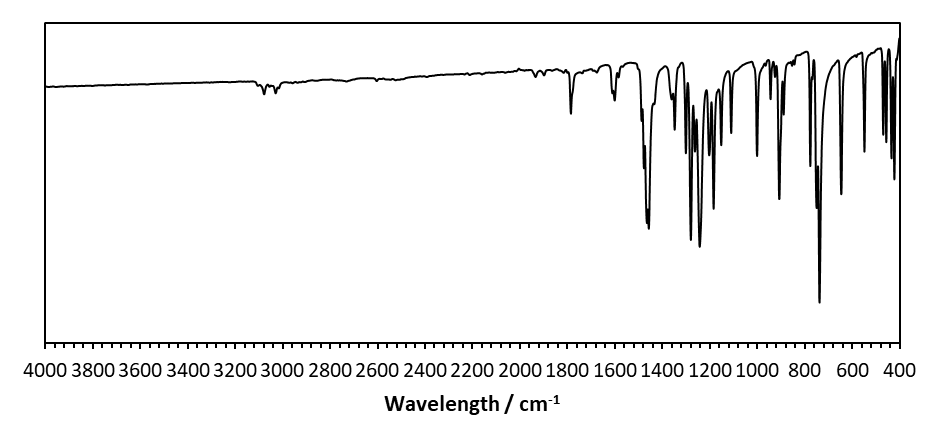


Scanning Electron Microscopy


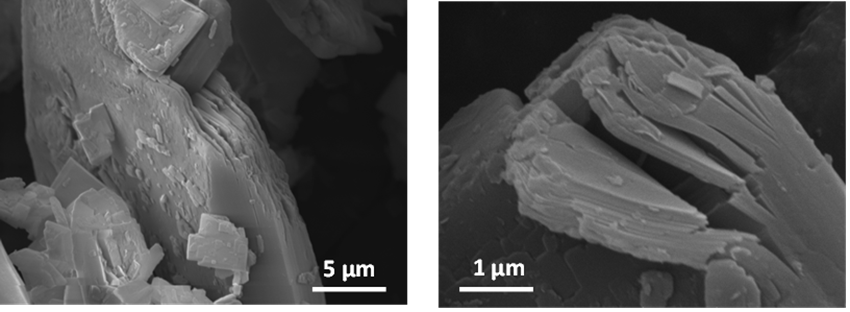


Figure S3: SEM of synthesised ZIF-7-III bulk MOF

TGA


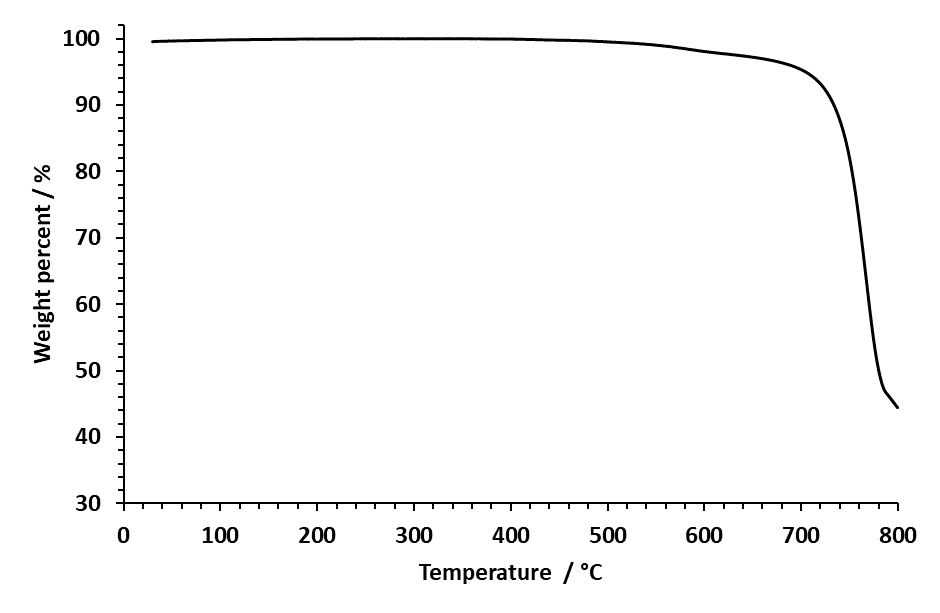


Figure S4: TGA plot for ZIF-7

1. Synthesis of ZIF-7-NH_2_-III MOF

Zinc nitrate hexahydrate (755 mg, 2.54 mmol), (962 mg, 8.14 mmol) benzimidazole and (1.08 g, 8.11 mmol) 5-aminobenzimidazole were dissolved in DMF (125 mL, 1.63 mol). After stirring for 1 h, the solution is kept statically at 30 °C for 2 weeks. ZIF-7 nanoparticles were then collected by centrifugation and thoroughly washed with methanol and dried at 50 °C overnight. Then subsequently dried at 120 C for 48 h in a vacuum oven. The obtained ZIF-7-I was redispersed in distilled water at a concentration of 0.5 wt % and then refluxed at 100 C for 1 week. The turbid mixture was washed with distilled water once and methanol three times, and then dried at 50 C overnight to obtain ZIF-7-III (436 mg, 0.694 mmol, 27 %) as a purple. Elemental Analysis %: C 56.1, H 3.3, N 18.7 Found Mass %: C 56.21, H 3.51, N 18.6; (ESI-NEG):[M]^+^ 134.1 (Bim-NH_2_), [M]^+^ 119.1 (Bim). 1Η ΝΜR (DCl/DMSO-d_6_): 9.73 (s, 1Η, **Bim-NH_2_**), 9.66 (s, 1Η, **Bim**), 8.03 (d, 1Η, **Bim-NH_2_**), 8.00 (s, 1H, **Bim-NH_2_**), 7.92 – 7.83 (m, 2Η, **Bim**), 7.64 (d, *J* = 2.0 Hz, 1Η, **Bim-NH_2_**), 7.63 – 7.55 (m, 2Η, **Bim**). Phase purity confirmed by XRPD with a comparison to the CCDC (675375) powder pattern for ZIF-7-III.

1. Characterisation of ZIF-7-NH_2_-III MOF

NMR


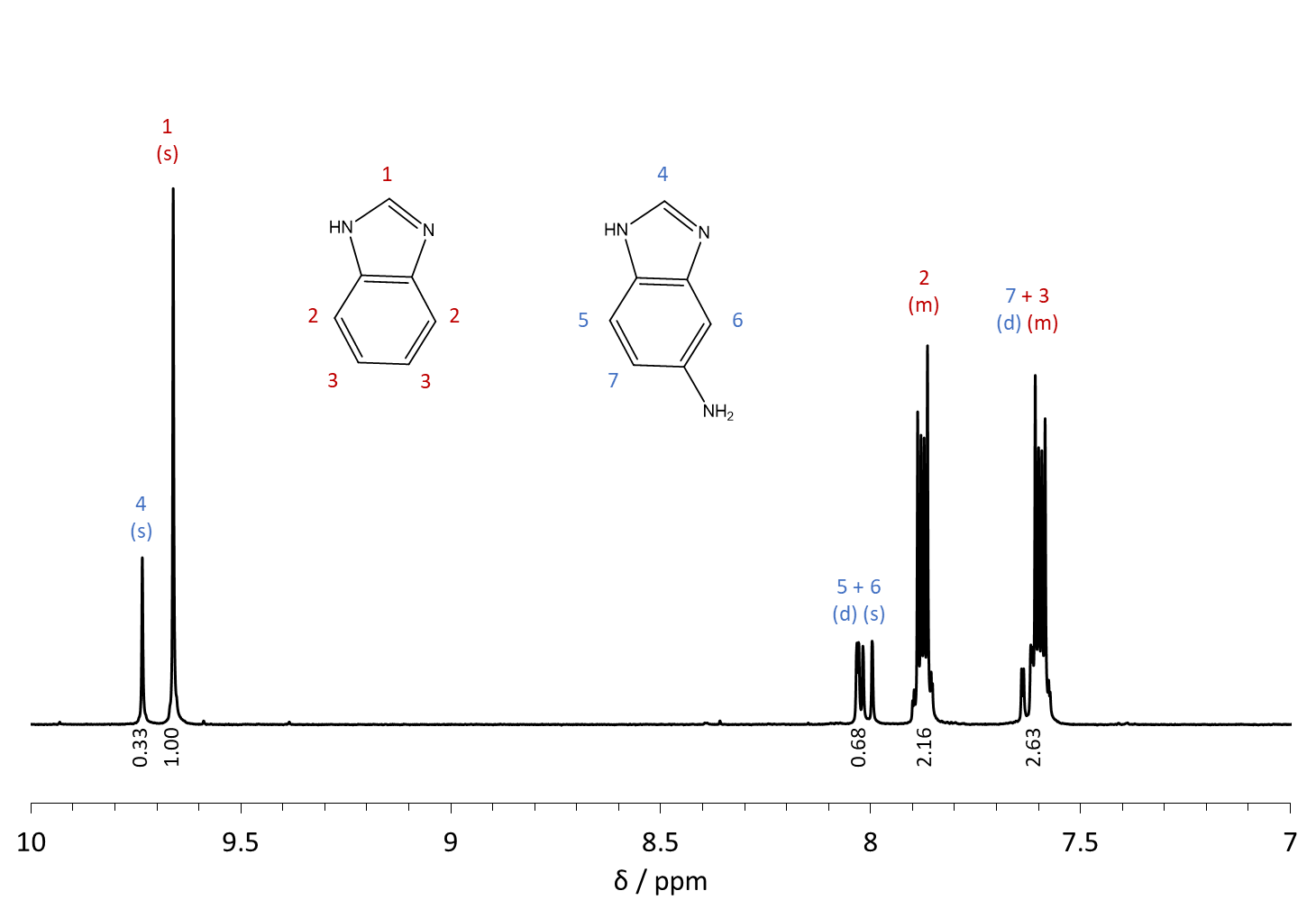


Figure S5: ^1^H NMR spectrum of ZIF-7-NH_2_ digested with DCl/DMSO-d_6_, with peaks assigned according to the inset molecular structures.

XRPD


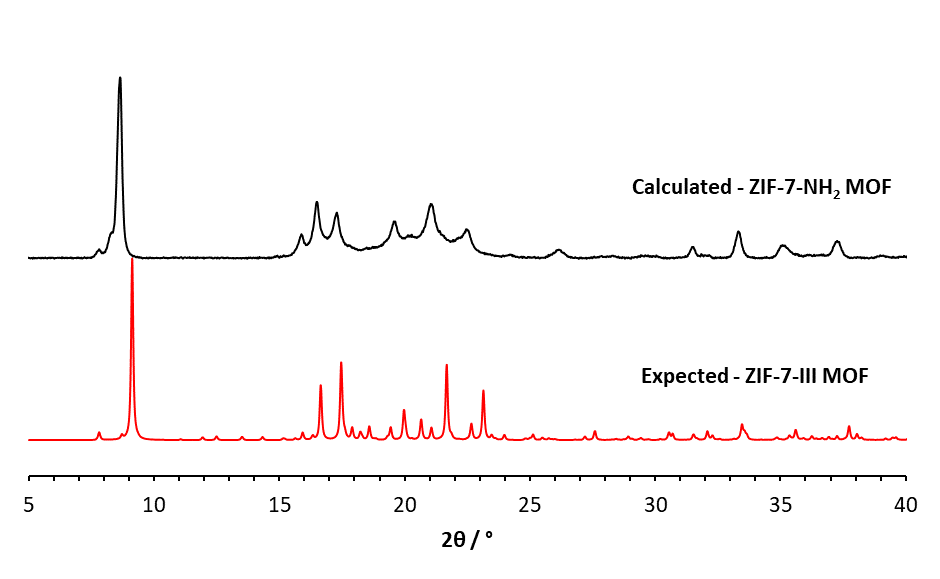


Figure S6: XRPD of synthesised ZIF-7-NH_2_ (black) with comparison to the expected pattern found in CCDC (675375) (red).


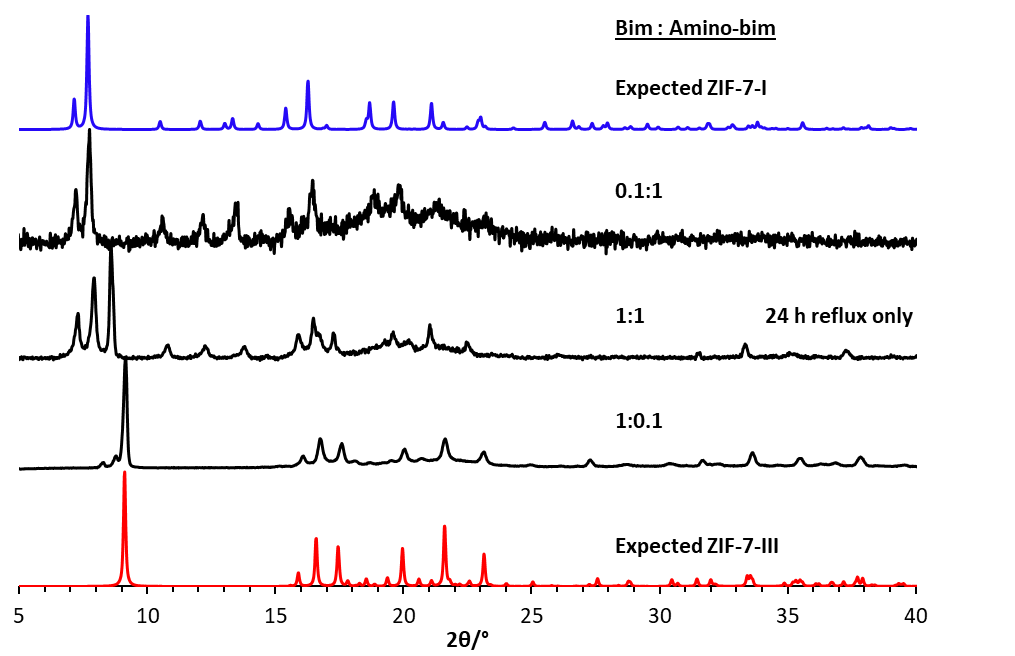


Figure S7: XRPD of synthesised ZIF-7-NH_2_ with different ratio of benzimidazole and 5-amino-benzimidazole added (black) compared to ZIF-7-III (red) and ZIF-7-I (blue).

Figure S8: XRPD of synthesised ZIF-7-NH_2_ (black top) with comparison to synthesised ZIF-7 (black bottom) the expected pattern found in CCDC (675375) (red) with inserted zoom of section 8-10 ° 2θ to show shift in [002].


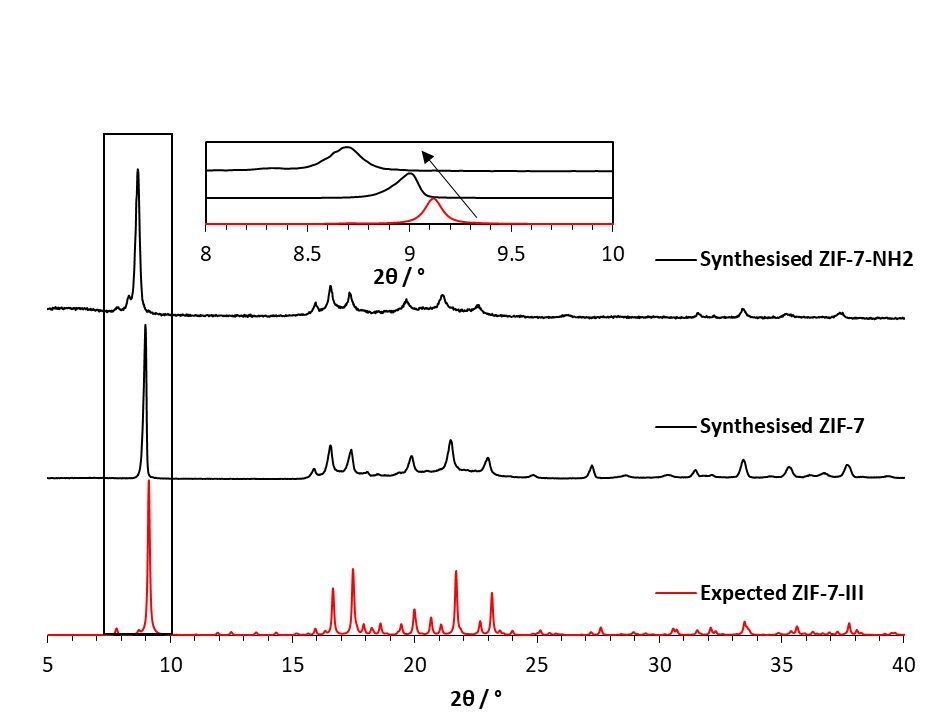


ATR-IR


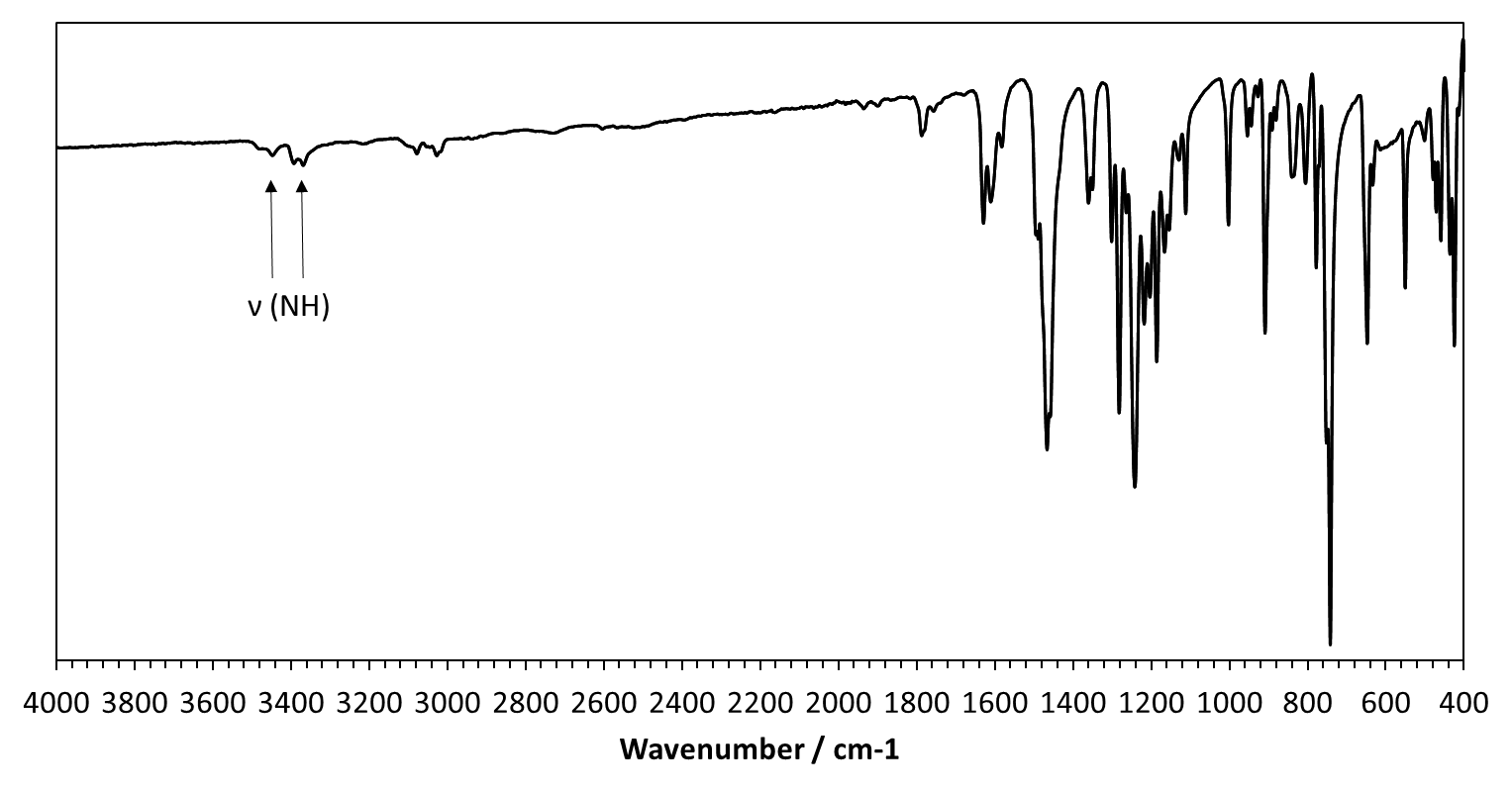


Figure S9: ATR-IR spectrum of ZIF-7-NH_2_

SEM


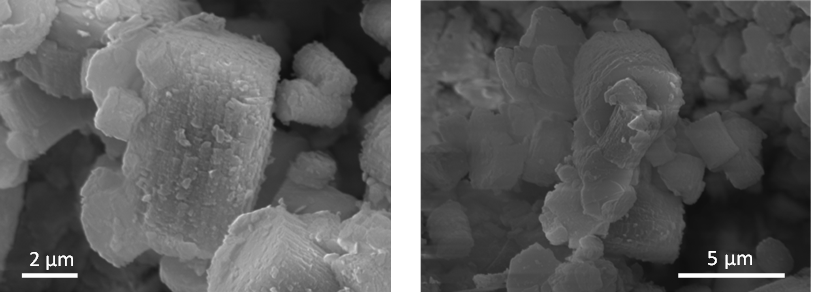


Figure S10: SEM of synthesised ZIF-7-NH_2_-III bulk MOF

TGA


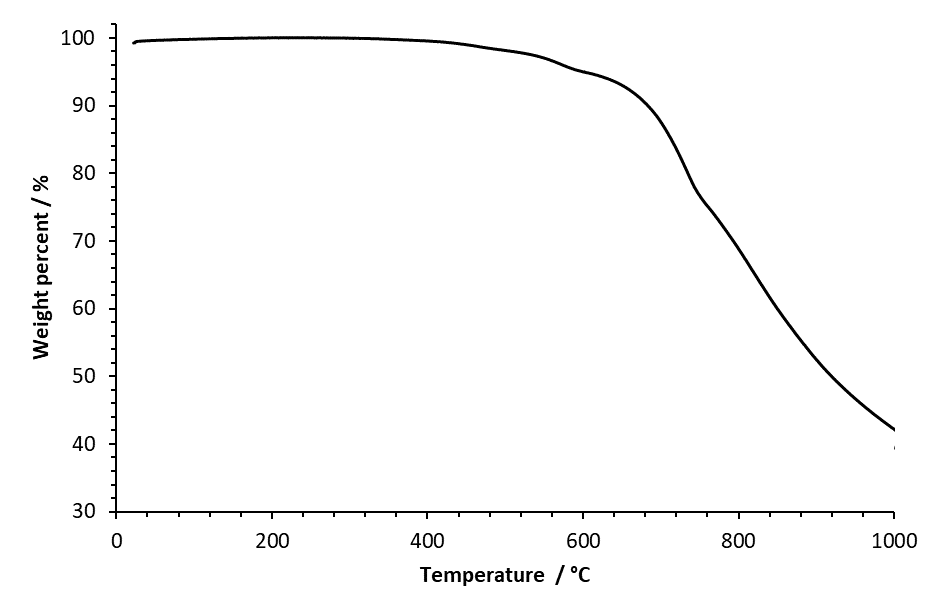


Figure S11: TGA plot for ZIF-7-NH_2_

1. Synthesis of Hf-BTB-NH_2_

To prepare Hf-BTB-NH_2_ MONs, hafnium chloride (34.59 mg, 0.108 mmol) and 2,4,6-Tris(4-carboxyphenyl)aniline (32.87 mg, 0.072 mmol) were added to a 20 mL screw-cap glass vial. DMF (12.5 mL) was added and the mixture ultrasonically dissolved to obtain a clear solution. Formic acid (2.5 mL) and H_2_O (0.7 mL) were added, the vial sealed and placed in a 120 °C oven for 48 h. The vial was taken out of the oven and cooled to room temperature. The turbid solution was centrifuged to collect Hf-BTB-NH_2_ as a pale-yellow precipitate. The precipitate was washed multiple times with DMF, followed by ethanol. To prevent the stacking of the MON layers, Hf-BTB-NH_2_ was kept in ethanol until needed for further studies. For analysis purposes the solid was collect via centrifugation and dried 120 °C overnight. Elemental Analysis %: C 27.3, H 2.04, N 1.18 Found Mass %: C 24.8, H 2.9, N 1.0. ^1^Η ΝΜR (ΝaOD/D_2_Ο, 24 hr sonication): 8.24 (s, 2Η), 7.72 – 7.65 (m, 4Η), 7.58 (d, *J* = 8.4 Hz, 2H), 7.21 (d, *J* = 8.3 Hz, 2Η), 7.11 (d, *J* = 8.2 Hz, 4Η), 7.03 (s, 2Η). Phase purity confirmed by XRPD with a comparison to NUS-8-Hf found in CCDC (1567189).

1. Characterisation of Hf-BTB-NH_2_

NMR

Figure S12: ^1^H NMR spectrum of Hf-BTB-NH_2_ digested with NaOD/D_2_O and a 24 hr sonication, with peaks assigned according to the inset molecular structure of BTB-NH_2_.


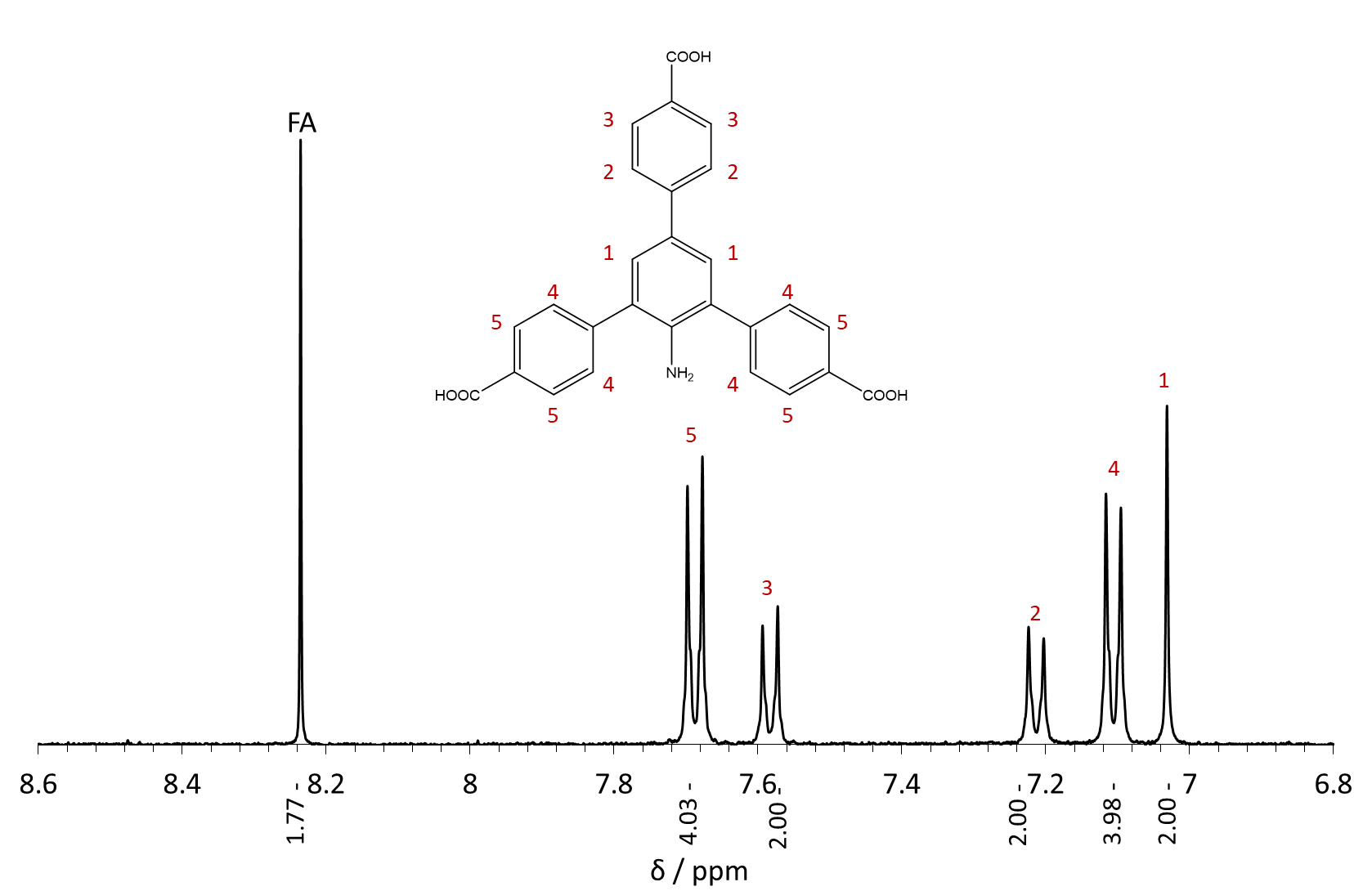


XRPD

Figure S13: XRPD of synthesised Hf-BTB-NH_2_ (black) with comparison to the pattern for NUS-8-Hf found in CCDC (1567189) (red). Insert of XRPD patterns of Zr-BTB-NH_2_ (red) taken from paper by Pu *et al..*^1^


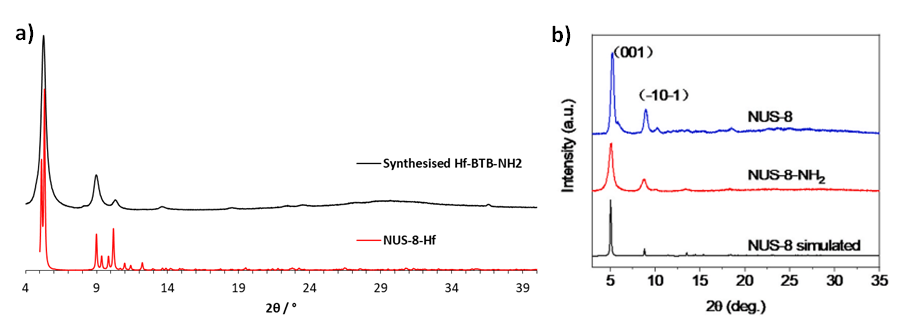


ATR-IR


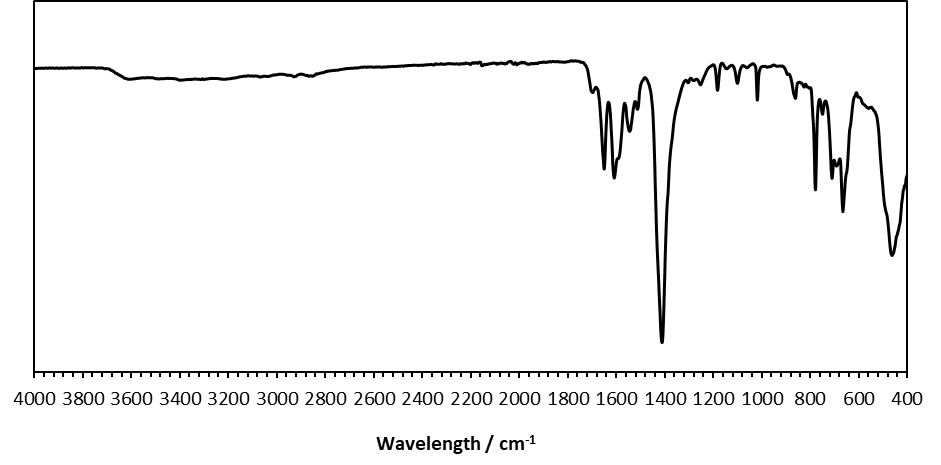


Figure S14: ATR-IR spectrum of Hf-BTB-NH_2_

SEM


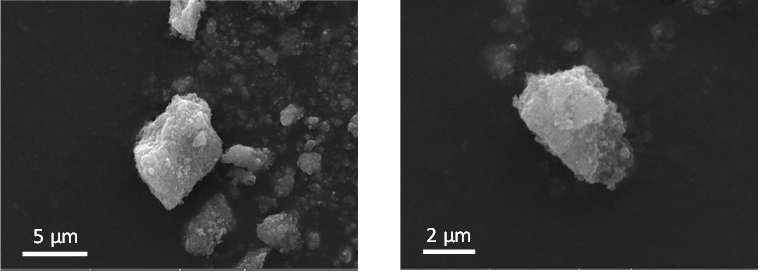


Figure S15: SEM of synthesised Hf-BTB-NH_2_ bulk MOF

TGA


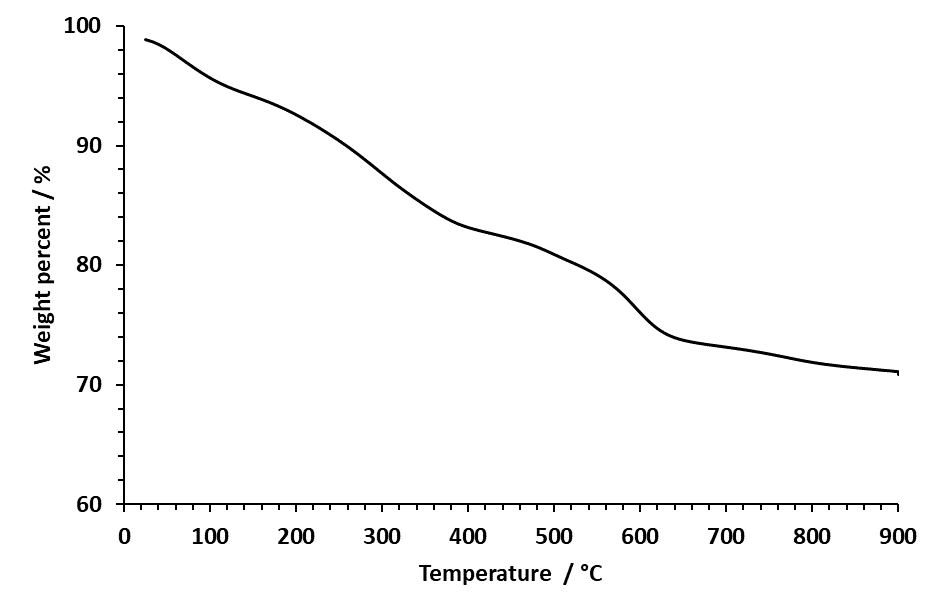


Figure S16: TGA plot for Hf-BTB-NH_2_

MON Synthesis and Characterisation

1. Exfoliation of ZIF-7 and ZIF-7-NH_2_ to access nanosheets

80 mg ZIF-7-III/ZIF-7-NH_2_-III was ultrasonicated in 120 mL of a mixed solvent of methanol and n-propanol (1:1, v/v) at 80 KHz for 2 h to access nanosheets. After being centrifuged at 500 rpm for 5 min, the collected supernatant containing the nanosheets is the centrifuged at 12,000 30mins.

1. Exfoliation of Hf-BTB-NH_2_ to access nanosheets

6 mg Hf-BTB-NH_2_ was ultrasonicated in mL of water at 37 KHz for 12 h to access nanosheets. To prevent the stacking of the MON layers, Hf-BTB-NH_2_ was kept in ethanol until needed for further studies.

1. Characterisation of ZIF-7, ZIF-7-NH_2_ and Hf-BTB-NH_2_ nanosheets

Tyndall Scattering


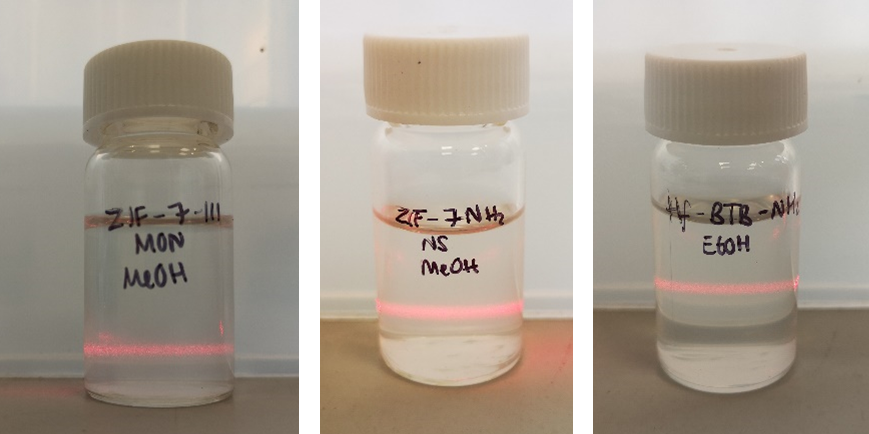


Figure S17: Tyndall scattering image of suspensions of ZIF-7, ZIF-7-NH_2_ and Hf-BTB-NH_2_

Atomic Force Microscopy

**ZIF-7**

Figure S18: AFM images and associated height profiles of ZIF-7 nanosheets


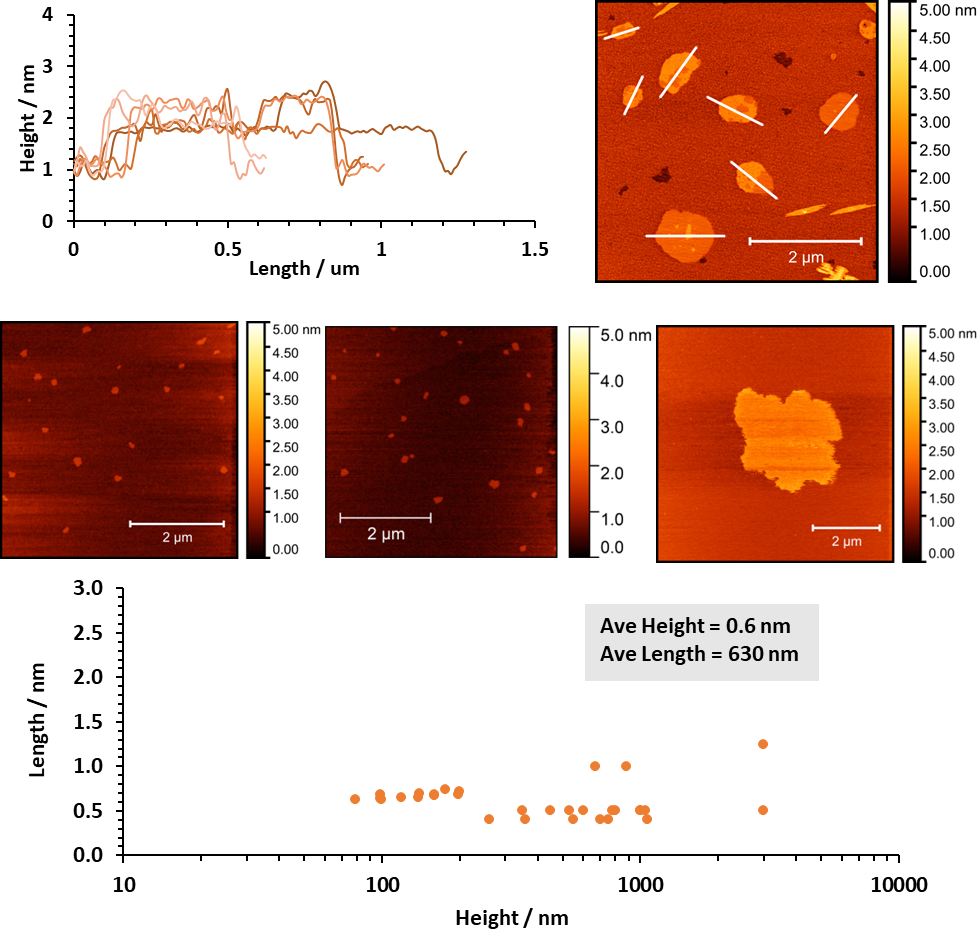


**ZIF-7-NH_2_**

Figure S19: AFM images and associated height profiles of ZIF-7-NH_2_ nanosheets


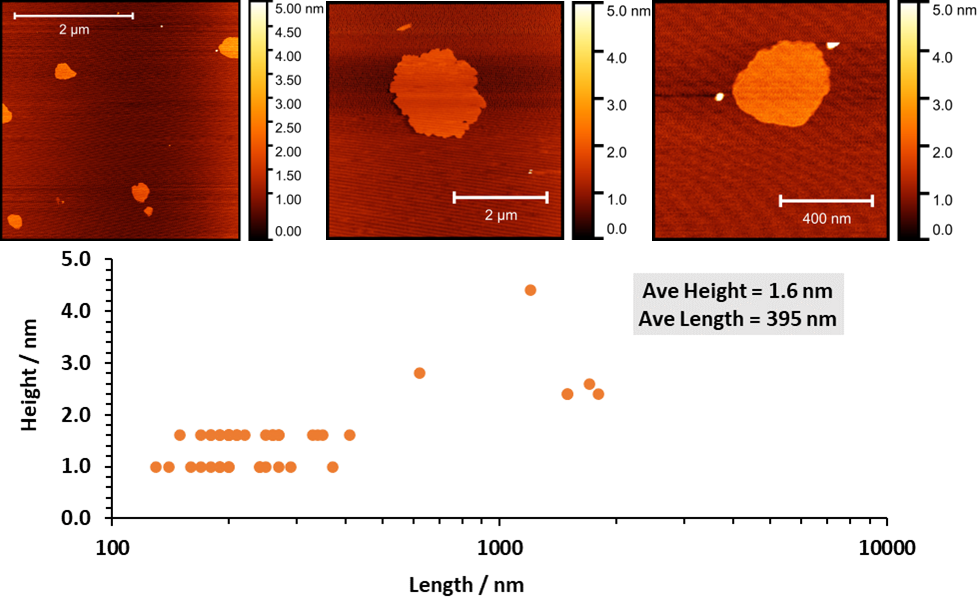


**Hf-BTB-NH_2_**

Figure S20: AFM images and associated height profiles of Hf-BTB-NH_2_ nanosheets


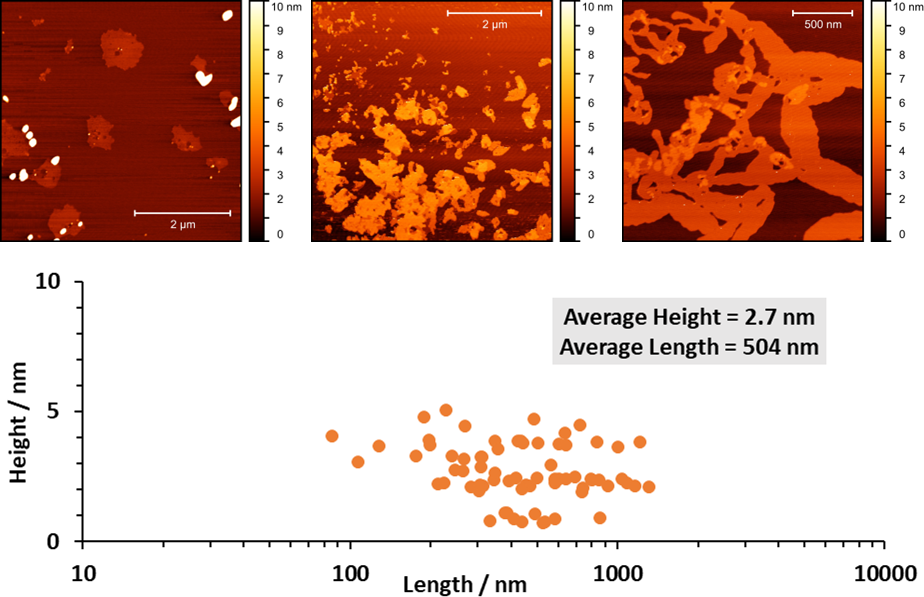


Dynamic Light Scattering


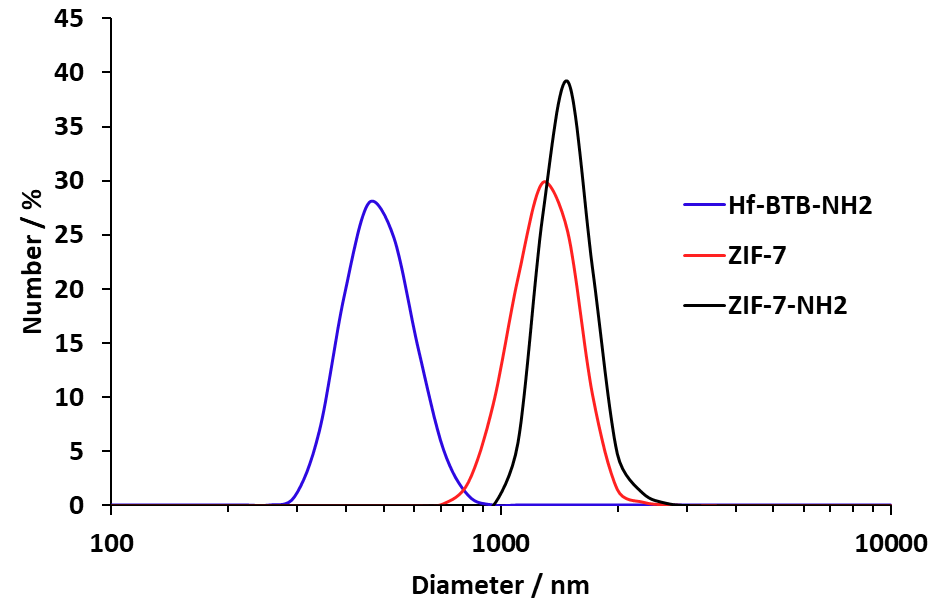


Figure S21: DLS number plot for MON suspensions of ZIF-7 (red) and ZIF-7-NH_2_ (black) in methanol and Hf-BTB-NH_2_ in ethanol.

Zeta Potential

Table S1: Zeta potential data obtained using the Smoluchowski method.

| **Sample Name** | **Temperature (°C)** | **Zeta Potential (mV)** | | **Zeta Deviation (mV)** | **Mobility (µmcm/Vs)** | **Conductivity (mS/cm)** |
| --- | --- | --- | --- | --- | --- | --- |
| ZIF-7 1 | 20 | -28.8 | | 8.3 | -2.043 | 0.206 |
| ZIF-7 2 | 20 | -30.5 | | 7.47 | -2.161 | 0.206 |
| ZIF-7 3 | 20 | -30.9 | | 9.29 | -2.194 | 0.207 |
|  | **20** | **-30.1** | **8.35** | | **-2.13** | **0.21** |
| ZIF-7-NH_2_ 1 | 20.1 | 2.22 | | 6.09 | 0.1574 | 0.246 |
| ZIF-7-NH_2_ 2 | 20 | 2.51 | | 6.05 | 0.1778 | 0.247 |
| ZIF-7-NH_2_ 3 | 20 | 2.11 | | 3.88 | 0.1493 | 0.248 |
|  | **20** | **2.3** | **5.34** | | **0.16** | **0.25** |
| Hf-BTB-NH_2_ 1 | 20 | -36.9 | | 7.95 | -2.615 | 0.231 |
| Hf-BTB-NH_2_ 2 | 20 | -37.2 | | 7.04 | -2.636 | 0.234 |
| Hf-BTB-NH_2_ 3 | 20 | -36.7 | | 6.43 | -2.605 | 0.236 |
|  | **20** | **-36.9** | **7.14** | | **-2.62** | **0.23** |

Contact Angle

Figure S22: Contact angle data for ZIF-7, ZIF-7-NH_2_ and Hf-BTB-NH_2_.


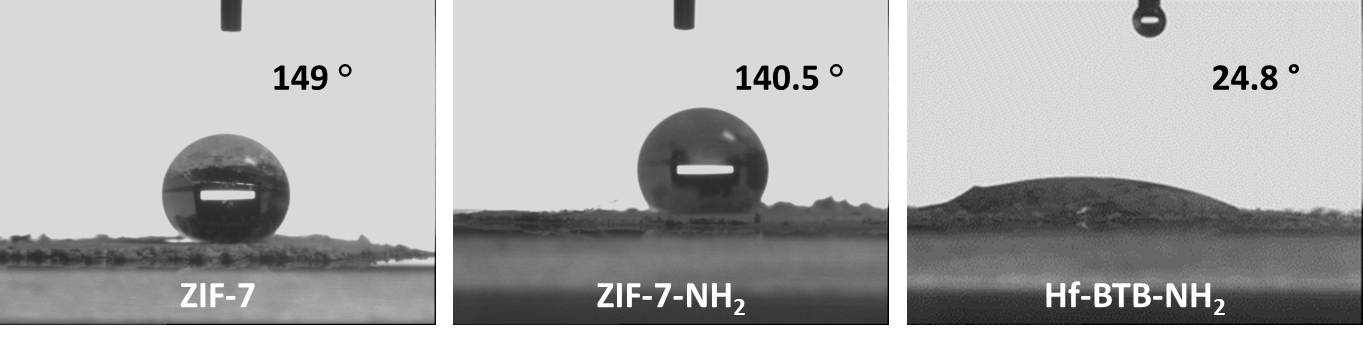


PBS Stability Test

**XRPD**

**ZIF-7**

Figure S23: XRPD pattern of ZIF-7-III before (black) and after (grey) exposure to PBS for 1 h and 24 h.


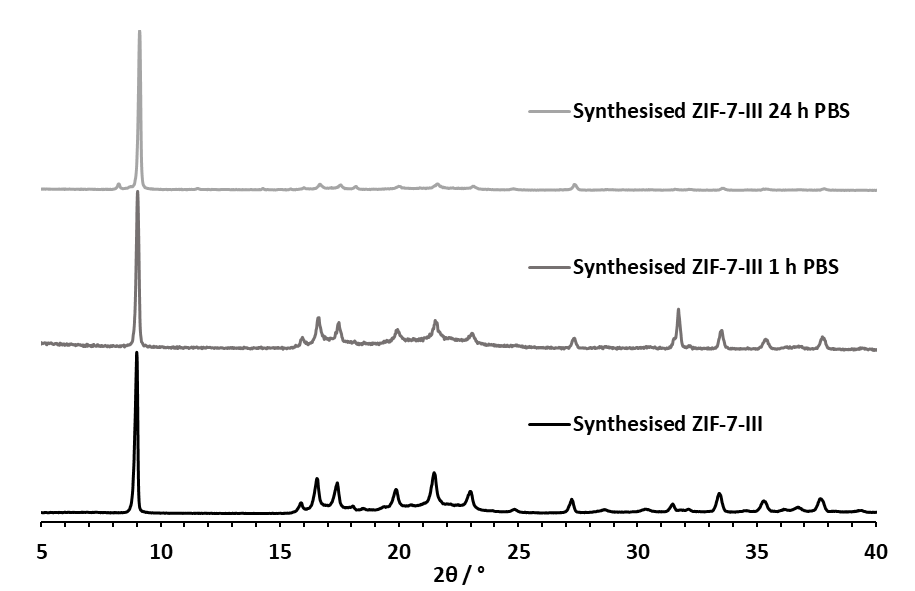


**ZIF-7-NH_2_**

Figure S24: XRPD pattern of ZIF-7-NH_2_ before (black) and after (grey) incubation with PBS for 1 h and 24 h.


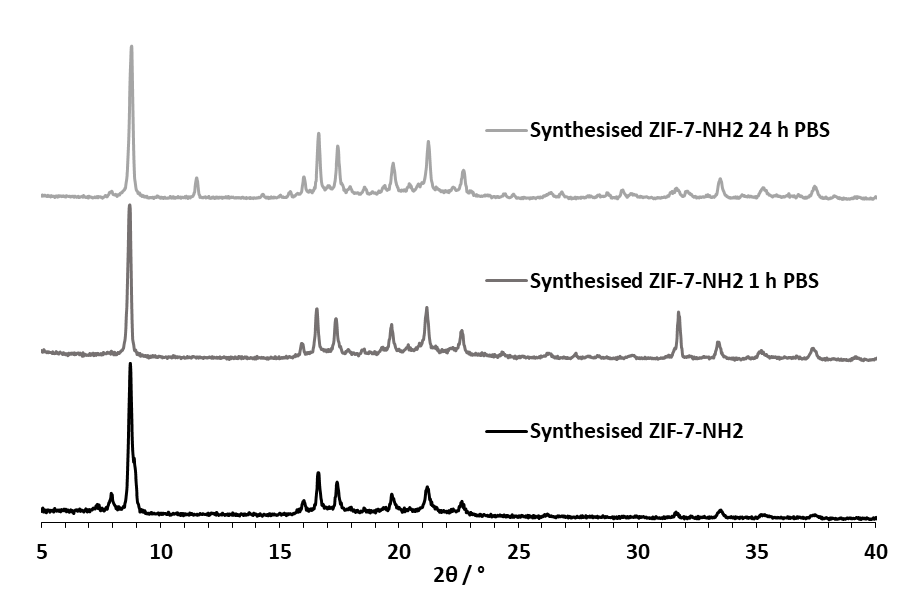


**Hf-BTB-NH_2_**

Figure S25: XRPD pattern of Hf-BTB-NH_2_ before (black) and after (grey) incubation with PBS for 1h and 24 h.


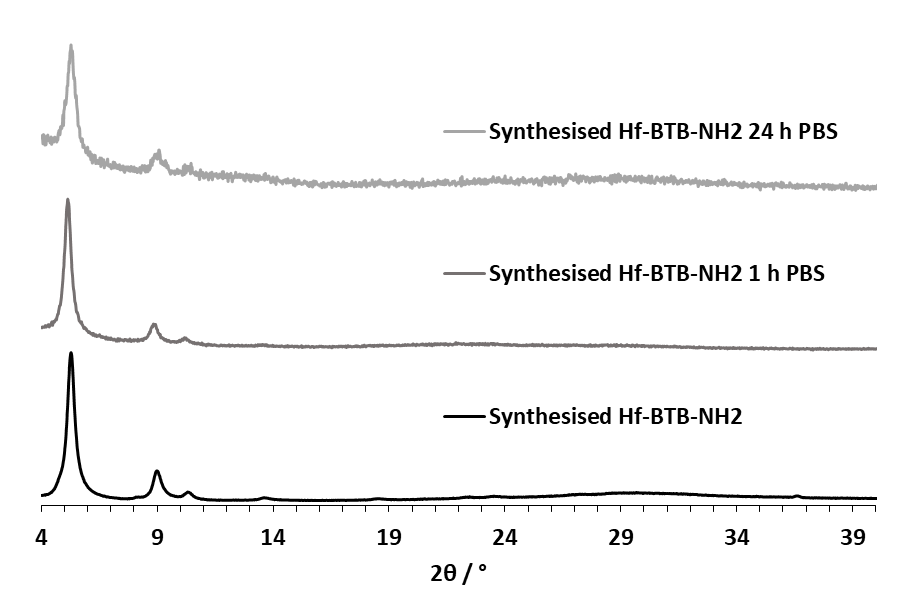


**AFM after PBS incubation**

Figure S26: AFM images and height profile of nanosheets of each system after incubation in PBS for 1 h.


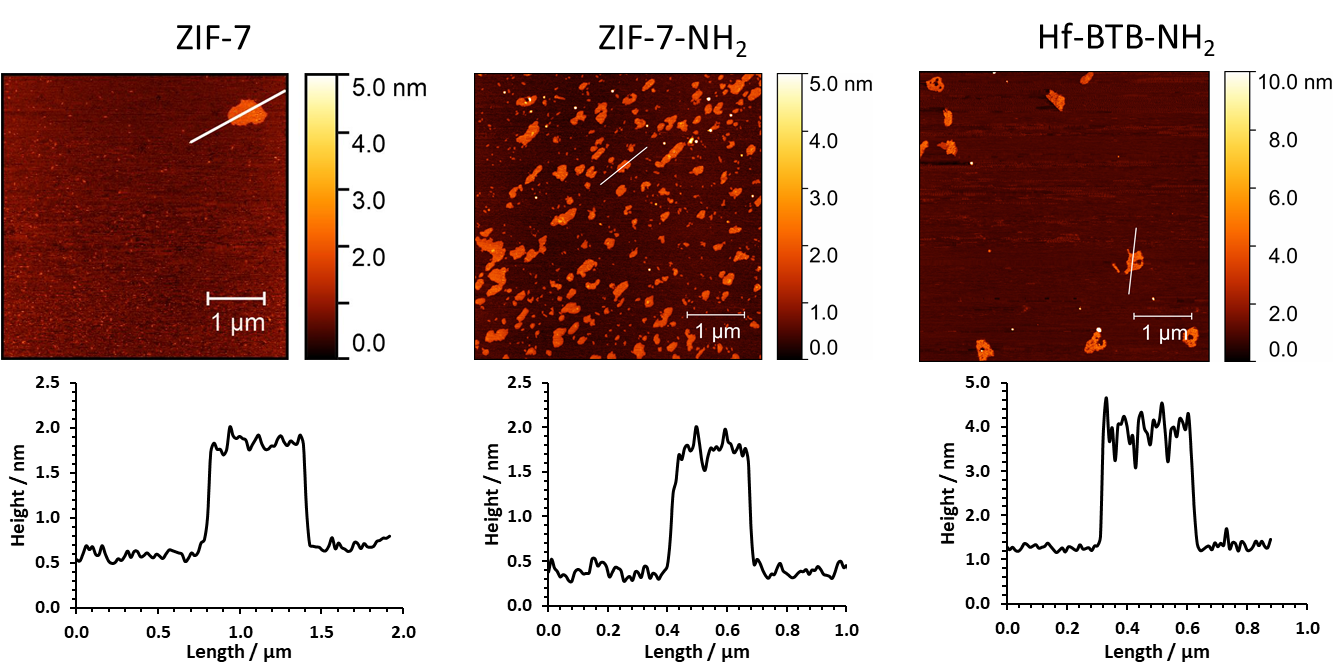


pH Stability Test

**ZIF-7**


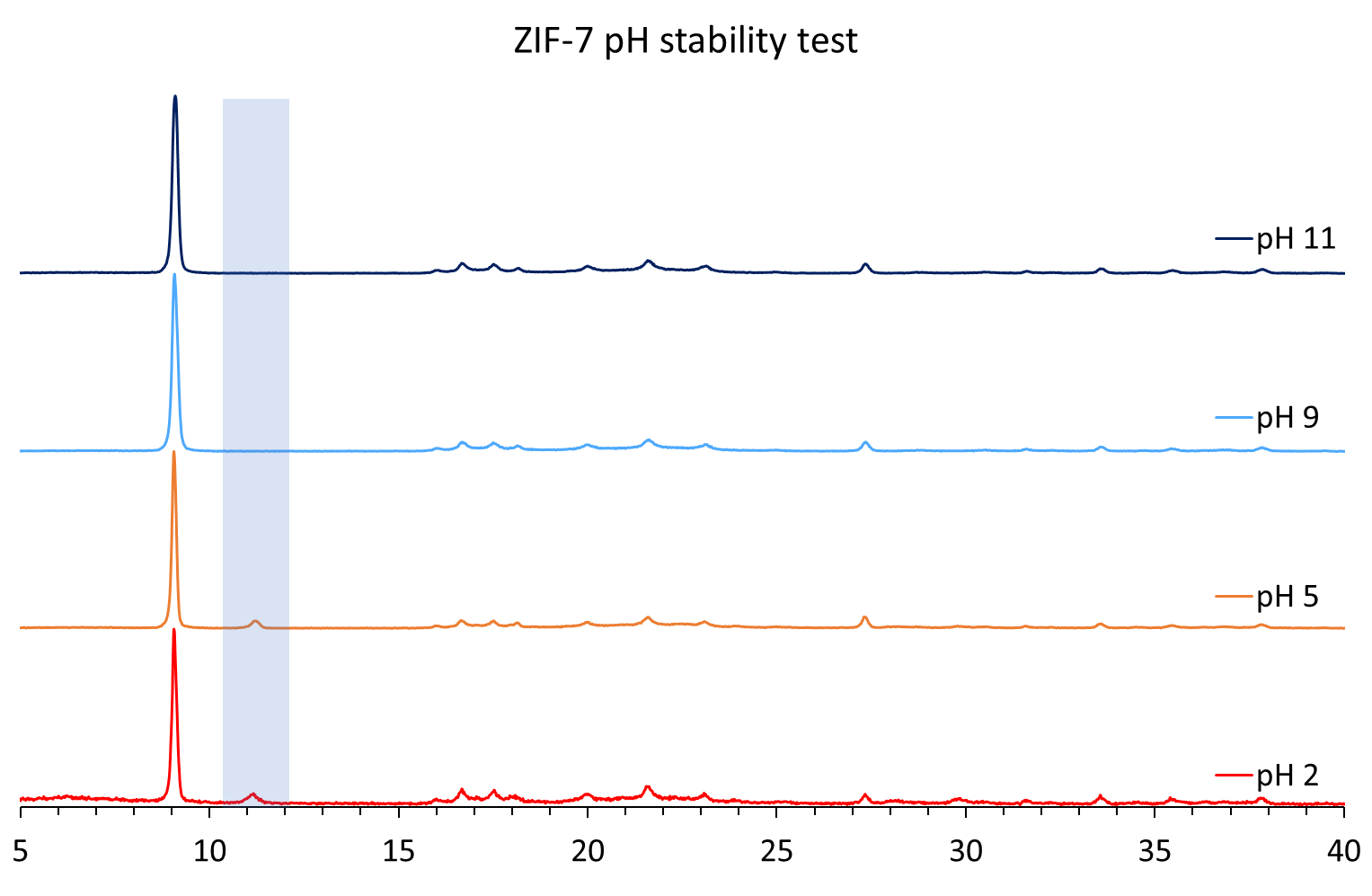


Figure S27: XRPD of ZIF-7 after incubation in aqueous solutions at different pHs


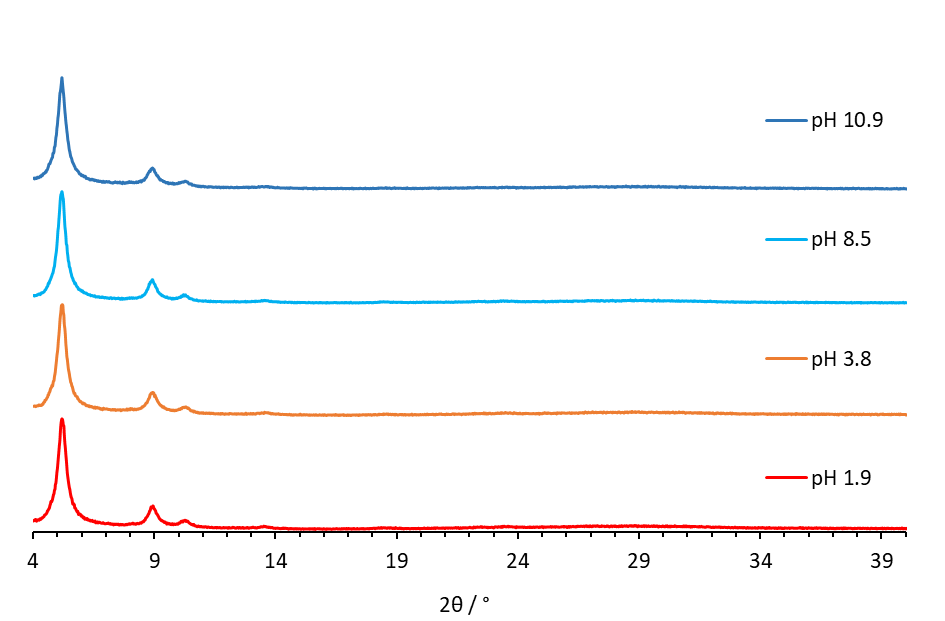


**ZIF-7-NH_2_**


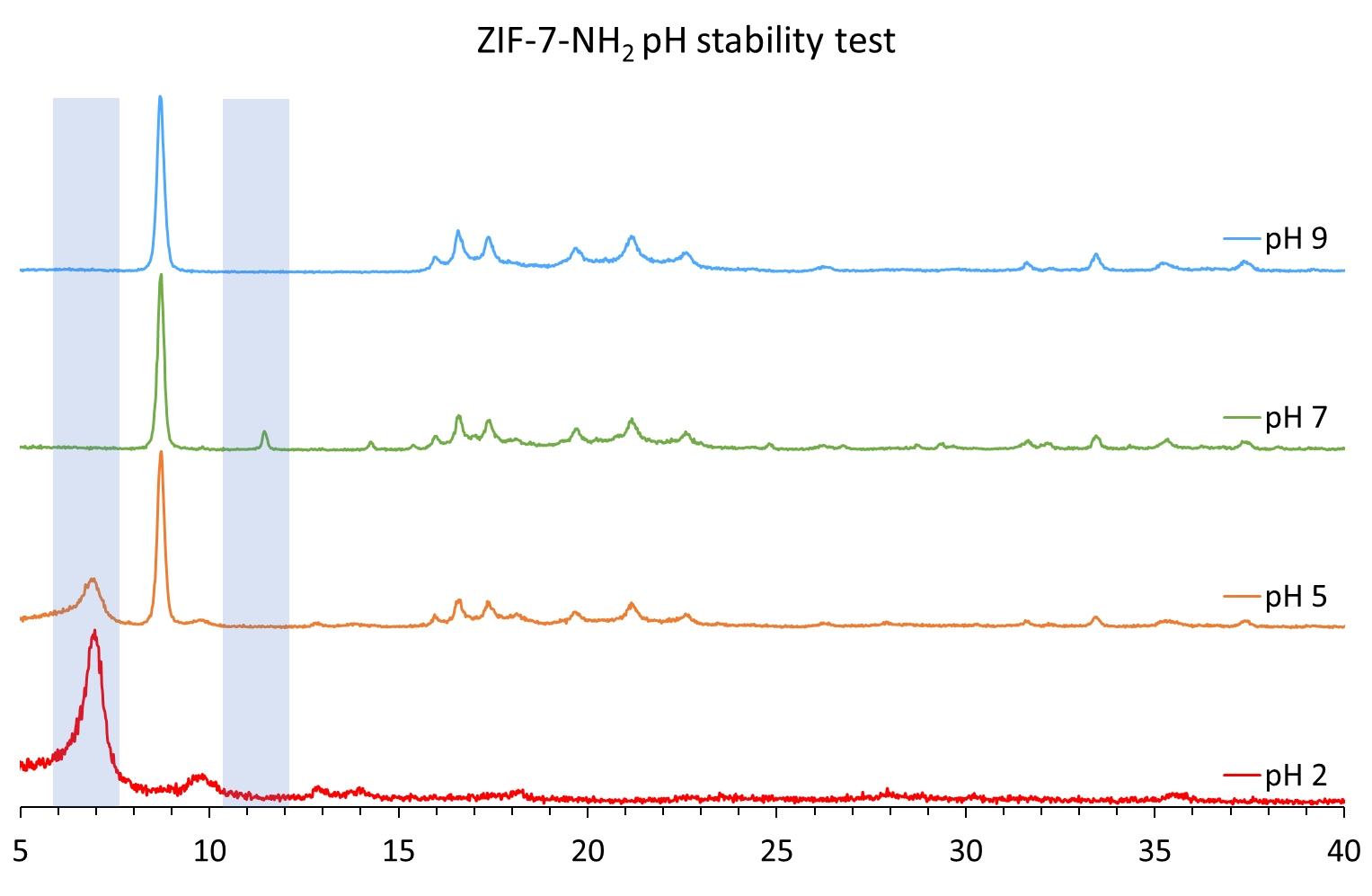


Figure S28: XRPD of ZIF-7-NH_2_ after incubation in aqueous solutions at different pHs


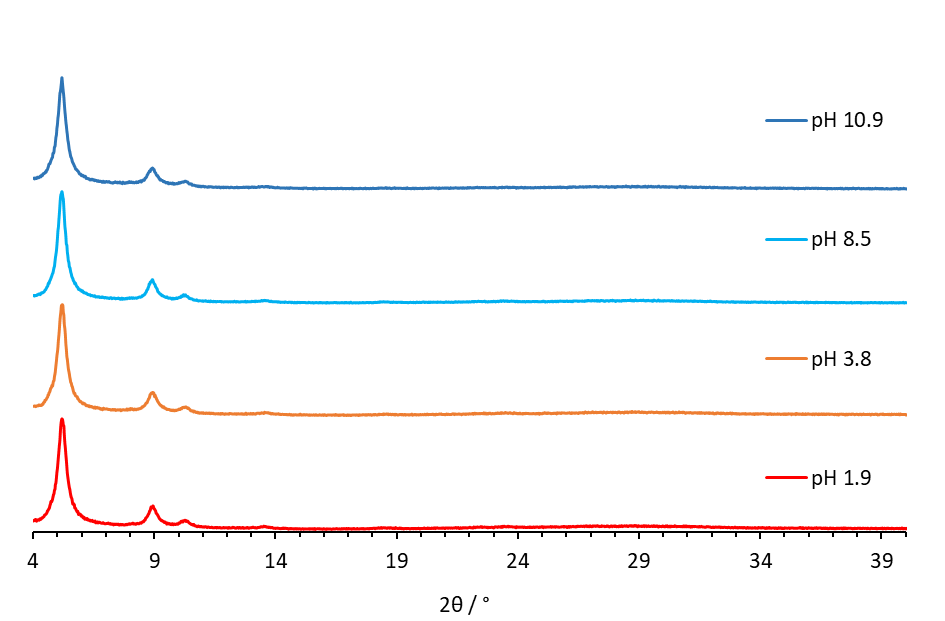


**Hf-BTB-NH_2_**


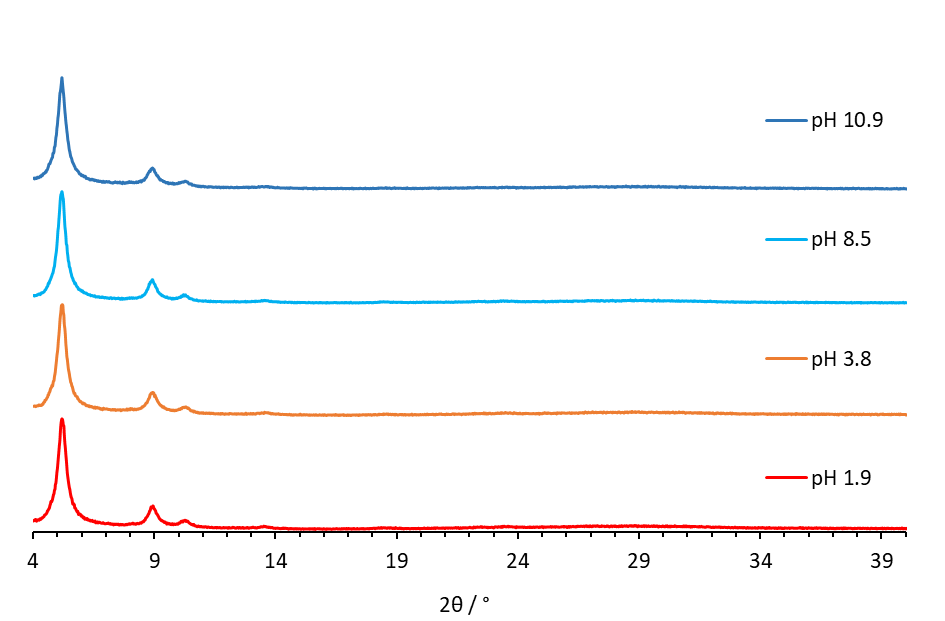


Figure S29: XRPD of Hf-BTB-NH_2_ after incubation in aqueous solutions at different pHs

Phage Display

MON binding peptides were isolated from a random 7-mer peptide library (New England Biolabs, Inc.) using the phage display protocol. The DNA sequences of the 7-mer peptides from the combinatorial peptide library are fused to the gIII of the bacteriophage M13. The peptides are separated from the pIII major coat protein by a short spacer sequence (GGGS). The peptide library has complexities on the order of 10^13^ independent clones, which is sufficient to encode the 1.28 x 10^9^ possible 7-mer peptide sequences. General phage methods were conducted according to the manufacturer’s recommendations.

MONs were incubated with the phage library in 2x blocking buffer (BB) phosphate buffered saline for 1 h on a rotating blood wheel. The particles were washed three times with PBS to remove unbound phage. After each wash, the frameworks were isolated by centrifugation (relative centrifugal force (RCF) 10 000 × g) for 10 min, and the supernatant was discarded. Bound phage were eluted with 0.2 M glycine pH 2.2, followed by trimethylamine. This resulted in MON digestion for ZIF-7 and ZIF-7-NH_2_ so it is assumed that all bound phage were eluted. The eluted phage were amplified in E. coli (ER2738) and purified by poly(ethylene glycol)-8000/sodium chloride (PEG/NaCl) precipitation with subsequent centrifugation steps. In subsequent biopanning rounds, at least 5 × 10^10^ phage were applied to a fresh solution of MONs in PBS. A total of three panning rounds were conducted to enrich the peptide pool with the highest affinity binders. For each MON system, 12 phage clones were analyzed by DNA sequencing.

1. Panning Round Data

Table S2: Panning round data for ZIF-7

| MON System | Panning round | Peptide Binding Sequence  (N’- C’ amidated) | Peptide Isoelectric Point / pH | Predicted overall charge in PD |
| --- | --- | --- | --- | --- |
| ZIF-7-NH_2_ | 3 | NNWWAPA | 14 | +1 |
|  | 1 | KMIDAFR | 11.39 | +2 |
|  | 1 | MRDHNLK | 11.39 | +2.1 |
|  | 1 | LWNPPNI | 14 | +1 |
|  | 1 | GSTMPIT | 14 | +1 |
|  | 1 | WIQQPFR | 14 | +2 |
|  | 1 | HAIFASN | 14 | +1.1 |
|  | 1 | SSPLGMA | 14 | +1 |

| MON System | Panning round | Peptide Binding Sequence  (N’- C’ amidated) | Peptide Isoelectric Point / pH | Predicted overall charge in PD |
| --- | --- | --- | --- | --- |
| ZIF-7 | 3 | YNYRNLL | 10.15 | +2 |
|  | 1 | TTFNLYP | 9.53 | +1 |
|  | 1 | LTNHIDM | 7.81 | +0.1 |
|  | 1 | GSWEHTT | 7.81 | +0.1 |
|  | 1 | TAKPIRE | 11.39 | +2 |
|  | 1 | SERLRTT | 12.1 | +2 |
|  | 1 | KVIELFS | 9.91 | +1 |
|  | 1 | EQLAVPL | 6.87 | +0 |
|  | 1 | MLPRQMT | 14 | +2 |
|  | 1 | WTRTVAN | 14 | +2 |

Table S3: Panning round data for ZIF-7-NH_2_

1. Zeta Potential

Peptide binding study – zeta potential. Hf-BTB-NH_2_ solution (1 mg/ml) in diluted PBS (1.37 mmol wrt. NaCl) was incubated for 1 h with various concentrations of the three identified binding peptides. Zeta potentials were determined after 1 h of incubation to examine any change in this parameter for the various peptides. The equation for K_d_ is provided in section 4.1 of the Supporting Information.

Figure S30: Change in Zeta Potential for each MON system on incubation with their phage display identified peptide (YNYRNLL - ZIF-7, NNWWAPA – ZIF-7-NH_2_, FTVRDLS – Hf-BTB-NH_2_)


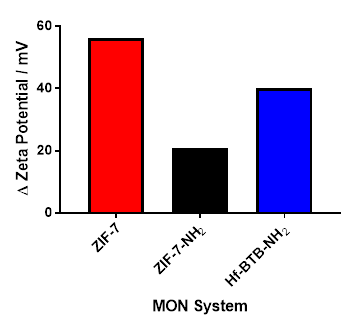


Figure S31: Zeta potential change when Hf-BTB-NH_2_ is incubated with the peptides YNYRNLL (black), NNWWAPA (red) and FTVRDLS (blue)


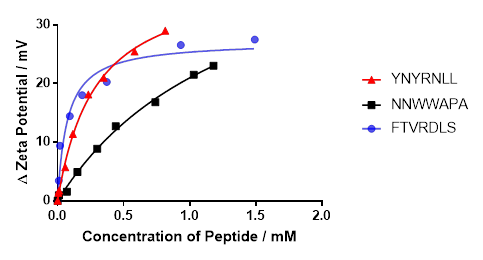


1. QCM binding studies

Preparation of MON-based QCM sensors. Quartz crystal microbalance (QCM) sensors coated with a 50 nm silica overlayer (QSX 303, ∼5 MHz fundamental frequency) were purchased from Q-Sense (Sweden). Each sensor was cleaned according to the manufacturer’s instructions. This protocol involved (i) UV/O_3_ treatment for 15 min (Bioforce UV/O_3_ cleaner, ∼9 mW cm^–2^, λ = 254 nm), (ii) exposure to 2% w/w sodium dodecylsulfate solution for 30 min, (iii) copious rinsing with deionized water and drying under N_2_, (iv) a final UV/O_3_ treatment for 15 min.

The sensor was prepared by adding 10 x 10 μL of MON solution (1 mg/ml, ethanol) dropwise to the clean QCM substrate while spin coating (Ossila Spin Coater) at 3,000 rpm for 10 min. The thickness of the resulting MON coating was measured using AFM to be approximately 100 nm.

QCM measurements were performed using an openQCM NEXT instrument (Novatech Srl., Italy) equipped with a temperature-controlled cell connected to a Masterflex Digital Miniflex peristaltic pump (Cole-Parmer Instrument Company, UK). All experiments were conducted in PBS buffer (pH 7.4) and no measurements were undertaken until the sensor frequency exhibited a drift of less than 0.1 Hz min^−1^; this typically occurred within 1 h of filling the cell. Once a stable signal was obtained, the phage solutions (1 x 10^12^ PFU / mL) in PBS were passed through the cell at a constant flow rate of 0.018 mL min^−1^ (minimum flow volume = 0.20 mL).

The adsorbed amount can be calculated using various models.^64–66^ The simplest and most widely applied of these is based on the Sauerbrey equation, which relates the change in frequency, ∆*f*, directly to the change in adsorbed mass per unit area, *m*,

$$m=C \times\frac{\Delta f}{n}$$

where *C* is a sensitivity constant (−0.177 (mg∙m^−2^) × Hz^−1^), Δ*f* is the change in the resonant frequency (Hz), and *n* is the overtone number. The third harmonic (*n* = 3) was used to calculate the adsorbed amount to avoid experimental artifacts associated with the fundamental harmonic that may occur if the sample is imperfectly mounted on the sensor.^66–68^

1. SPR binding studies

**Immobilisation of nanosheets onto the SPR Sensor Surface.** Planar polyethylene glycol/carboxyl coated Au chips, purchased from Reichert Technologies (Buffalo, USA) were installed onto a Reichert 2 SPR following the manufacturer’s instructions (https://www.reichertspr.com/products/planar-polyethylene-glycol-carboxyl-sensor-chip). The surface of the chips used consist of a self-assembled monolayer consisting of polyethylene glycol-terminated alkanethiol (90%) and COOH-terminated alkanethiol (10%) with the COOH functionality providing an attachment site for covalently immobilizing molecules of interest. The sensor surface was preconditioned by running buffer PBST (PBS pH 7.4 and 0.01 % Tween 20) at 10 µL min^-1^ until a stable baseline was obtained. The flow rate of 10 µL was maintained throughout the immobilisation process. In order to activate carboxyl groups on the surface of the sensor chip, a freshly prepared aqueous solution (1 mL) of EDC (N-(3-dimethylaminopropyl)-N’-ethylcarbodiimide hydrochloride, 40 mg) and NHS (N-hydroxysuccinimide, 10 mg) was injected onto the sensor chip surface for 6 minutes. To the activated surface, 300 µg of the Hf-BTB-NH_2_ nanosheets dissolved in 1 mL of the running buffer (PBST) and 10 mM sodium acetate, was injected only to the left channel of the surface for 1 minute. Finally, quenching solution (1 M ethanolamine, pH 8.5) was injected for 8 minutes to deactivate carboxyl groups and wash away the unbound Hf-BTB-NH_2_ nanosheets. A continuous flow of running buffer (PBST) at 10 µL min^-1^ was maintained after Hf-BTB-NH_2_ nanosheet immobilisation. SPR assays were carried out after a stable baseline was achieved. The left channel was the working channel, and the right channel was the reference.Figure S32 shows the 3 step process used to imobilise the MON on the surface of a planar polyethylene glycol/carboxyl layered gold chip. Monolayer formation of MONs is expected as the EDC/NHS coupling chemistry used to covalently attach the amine functionalities on the surface of the MONs to the carboxylic acid functionalities on the SPR chip surface requires direct contact. Maximal coverage on the chip was achieved by adding the MONs in excess with a relatively slow flow whilst excess MON will be washed away to ensure monolayer formation.


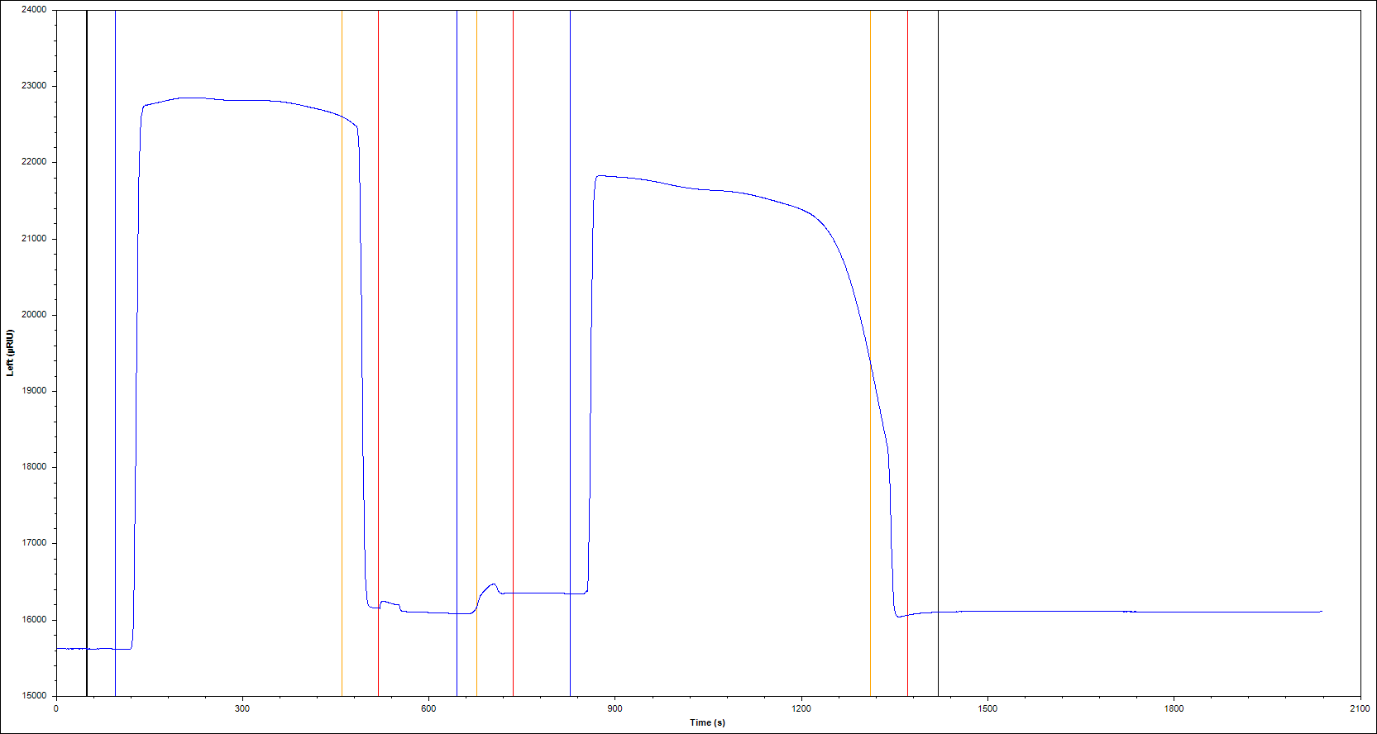
Figure S32. The immobilisation of the MON onto the surface of a planar polyethylene glycol/carboxyl layered gold chip: A. injection of EDC/NHS (dissolved in water) over the chip surface. B. injection of MON (dissolved in running buffer) over the chip surface. C, injection of a 1 M ethanolamine quenching solution over the chip surface. The difference between start and finish highlights deposition of MON.

B

C

A

**Kinetic Analysis Using SPR.** Kinetic analysis was initiated by injection of the running buffer PBST (blank) onto the Hf-BTB-NH_2_ immobilised sensor surface for 2 minutes, followed by PBST for 5 minutes. The binding kinetics of an individual Hf-BTB-NH_2_ nanosheet to the selected target was determined from serial dilutions (five concentrations, 4-64 nM) of the selected target under study. Each dilution was injected for 2 minutes (association) followed by PBST for 5 minutes (dissociation). After dissociation, the target was removed from the immobilized surface by injecting regeneration buffer (10 mM Glycine-HCl, pH 2) for 1 minutes followed by PBST for 1 minutes. The same procedures were repeated for the remaining four dilutions of the target. After the analyses were completed, signals from the left channel were subtracted from signals from their respective reference channel (the right channel).

The SPR responses from five concentrations of the target compound (4-64 nM) were fitted to a 1:1 bio-interaction (BI) mdel (Langmuir fit model) utilizing TraceDrawer software. Association rate constants (ka), dissociation rate constants (kd), and maximum binding (Bmax) were fitted globally, whereas the BI signal was fitted locally. The equilibrium dissociation constant (K_D_) was calculated from the ratio kd/ka.

Figure S33: Representative SPR sensorgrams of molecular interactions of the Hf-BTB-NH_2_ nanosheets immobilised on planar polyethylene glycol/carboxyl coated Au chips, to solutions containing five concentrations of target and non-target peptides. Target peptide (A) FTVRDLS and non target peptide (B) YNYRNLL) and (C) NNWWAPA

**Limit of detection (LOD)**

Using the maximum signal (µRIU) from the SPR fitted curves (Figure S31), a concentration calibration (Figure S33) was plotted to estimate the theoretical limit of detection as 0.39 nM (376 ng/L).

Figure S34: Plot of relative signal versus concentration of FTVRDLS (target peptide) binding to Hf-BTB-NH_2_ concentration calibration, used to calculate limit of detection for the SPE sensor.

1. Isoelectric point calculations

The net charge (*Z*) of a peptide at a certain pH can be estimated by calculating:

$$Z= \sum_{i} N_{i}\frac{{10}^{pKa_{i}}}{{10}^{pH}+{10}^{pKa_{i}}}-\sum_{j} N_{i}\frac{{10}^{pKa_{j}}}{{10}^{pH}+{10}^{pKa_{j}}}$$

where N_i_ are the number, and pKa_i_ the pKa values, of the N-terminus and the side chains of Arginine, Lysine, and Histidine. The j-index pertain to the C-terminus and the Aspartic Acid, Glutamic Acid, Cysteine, Tyrosine amino acids.

References

(1) Pu, Y.; Yang, Z.; Wee, V.; Wu, Z.; Jiang, Z.; Zhao, D. Amino-Functionalized NUS-8 Nanosheets as Fillers in PIM-1 Mixed Matrix Membranes for CO2 Separations. *J. Memb. Sci.* **2022**, *641*, 376–7388. https://doi.org/10.1016/j.memsci.2021.119912.
